# Integrative Multiomics Profiling Reveals Immune Determinants of Response to Disitamab Vedotin Plus Penpulimab in HER2‐Low Breast Cancer

**DOI:** 10.1002/mco2.70988

**Published:** 2026-10-04

**Authors:** Xiaoxiao Liu, Yuting Song, Lei Liu, Aaron Qi Zhang, Xin Xie, Bing Wei, Ping He, Chunying Zhuang, Juanjuan Li, Xi Yan, Tinglun Tian, Xiaorong Zhong, Jie Chen, Yan Cheng, Dan Zheng, Shibao Li, Hao Wang, Ting Luo

**Affiliations:** ^1^ Institute of Breast Health Medicine Sichuan Provincial Engineering Research Center of Intelligent Diagnosis and Treatment of Breast Diseases Breast Center Department of Medical Oncology Cancer Center West China Hospital Sichuan University Chengdu Sichuan China; ^2^ Department of Radiation Oncology Cancer Center Affiliated Hospital of Xuzhou Medical University Cancer Institute Xuzhou Medical University Xuzhou Jiangsu China; ^3^ Department of Physiology School of Basic Medicine and Life Sciences Xuzhou Medical University Xuzhou Jiangsu China; ^4^ Department of Laboratory Medicine Affiliated Hospital of Xuzhou Medical University Xuzhou Jiangsu China; ^5^ Department of Anatomical and Cellular Pathology State Key Laboratory of Translational Oncology The Chinese University of Hong Kong Hong Kong SAR China; ^6^ Department of Pathology West China Hospital Sichuan University Chengdu Sichuan China; ^7^ Division of Breast Surgery Department of General Surgery West China Hospital Sichuan University Chengdu Sichuan China; ^8^ Hepatobiliary Center of The First Affiliated Hospital Research Unit of Liver Transplantation and Transplant Immunology Chinese Academy of Medical Sciences Nanjing Medical University Nanjing Jiangsu China

**Keywords:** biomarker, disitamab vedotin, HER2‐low breast cancer, neoadjuvant therapy, pathologic complete response, penpulimab

## Abstract

This prospective single‐arm study evaluated neoadjuvant disitamab vedotin plus penpulimab in Stage II–III HER2‐low breast cancer (immunohistochemistry 1+ or 2+, fluorescence in situ hybridization‐negative), a population lacking targeted neoadjuvant options. Twenty patients received the combination every 3 weeks for six cycles before surgery. In the intention‐to‐treat population, the pathologic complete response (pCR) was 20.0% (four out of 20; 95% CI, 5.7–43.7%), and the objective response rate (ORR) was 45.0% (nine out of 20; 95% CI, 23.1–68.5%). Among 16 patients who completed protocol‐defined treatment and surgery, pCR was 25.0% (four out of 16; 95% CI, 7.3–52.4%), ORR was 56.3% (nine out of 16; 95% CI, 29.9–80.2%), and 31.3% achieved residual cancer burden Class 0–1. Grade ≥3 adverse events occurred in 25.0%; no treatment‐related deaths occurred. pCR‐associated tumors had a pretreatment immune‐active microenvironment enriched in inflammatory proteins, transcripts, and tumor‐infiltrating lymphocytes, whereas nonresponders displayed a higher M2/M1 macrophage ratio. An exploratory baseline score combining CCL19 and the M2/M1 ratio showed pCR/non‐pCR separation (AUC = 0.8889) but attenuated on internal validation (LOOCV AUC = 0.6667, optimism‐corrected bootstrap AUC = 0.8375). This combination showed preliminary antitumor activity with manageable safety; however, given the small sample size and wide confidence intervals, these findings preclude definitive conclusions about efficacy and warrant validation in larger randomized controlled trials.

## Graphical abstract

This prospective single‐arm study evaluated neoadjuvant disitamab vedotin plus penpulimab in 20 patients with Stage II–III HER2‐low breast cancer. The regimen showed preliminary activity (pCR 20.0% intention‐to‐treat, 25.0% evaluable; ORR 56.3%) with manageable toxicity. Integrated multiomics analyses linked response to a more immune‐active baseline microenvironment, lower M2/M1 polarization, and an exploratory BRPscore—hypothesis‐generating findings requiring validation in larger randomized trials.

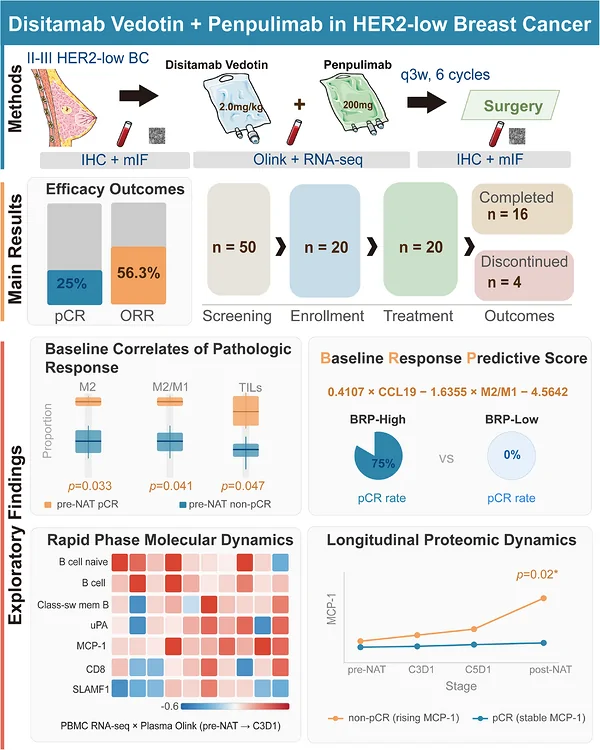

## 1 Introduction

Human epidermal growth factor receptor 2 (HER2)‐positive breast cancer is associated with aggressive biology, low sensitivity to chemotherapy, and poor prognosis [1]. The development of HER2‐targeted therapies has transformed outcomes for this subgroup [2]. Multiple targeted agents, including monoclonal antibodies, tyrosine kinase inhibitors, and antibody–drug conjugates (ADCs), have been approved for HER2‐positive breast cancer [1, 3, 4]. However, the therapeutic benefit of these approaches is limited in tumors with low HER2 expression, representing an unmet clinical need.

More than half of HER2‐negative metastatic breast cancers demonstrate low‐level HER2 expression, defined as immunohistochemistry (IHC) 1+ or IHC 2+ with in situ hybridization (ISH) negativity [5, 6]. HER2‐low tumors can be further classified by hormone receptor (HR) status into HR‐positive and triple‐negative breast cancer (TNBC) subtypes [7]. For HR‐positive disease, current management follows standard HR‐positive breast cancer protocols, with recommended neoadjuvant regimens including anthracycline–taxane chemotherapy or endocrine therapy; however, the overall pathologic complete response (pCR) rate remains below 10%. For HER2‐low TNBC, National Comprehensive Cancer Network (NCCN) guidelines recommend anthracycline–taxane combinations as the backbone of neoadjuvant therapy. Reported pCR rates range from 28 to 35% with combination therapy, compared with approximately 20% with anthracyclines alone and 12% with taxanes alone [8]. Addition of platinum increases pCR to about 50% but at the expense of greater toxicity and reduced tolerability [9, 10]. More recently, KEYNOTE‐522 and IMpassion031 showed that adding immune checkpoint inhibitors to chemotherapy significantly improved pCR and event‐free survival (EFS) [11, 12], and these regimens are now recommended for TNBC by the Chinese Society of Clinical Oncology guidelines. In China, however, approval of pembrolizumab in the neoadjuvant setting is restricted to its use with chemotherapy in patients with high‐risk early TNBC and a tumor programmed death‐ligand 1 (PD‐L1) combined positive score (CPS) of ≥20. There is therefore an important unmet clinical need for effective and well‐tolerated neoadjuvant options for the broader HER2‐low population.

ADCs combine a monoclonal antibody, a chemical linker, and a cytotoxic payload, thus uniting target specificity with potent cell killing [13]. Encouraging clinical results have been reported with several ADCs in HER2‐low breast cancer, including DS‐8201 (T‐DXd), disitamab vedotin (RC48), and sacituzumab govitecan [5, 14, 15, 16, 17]. RC48 is composed of a fully humanized anti‐HER2 antibody linked to the microtubule inhibitor monomethyl auristatin E (MMAE) via a protease‐cleavable vedotin linker [18, 19]. Preclinical studies have shown that RC48 has a higher affinity for immobilized HER2 than trastuzumab [18]. When RC48 binds to HER2 on tumor cells, it is internalized and MMAE is released through proteolytic cleavage of the valine‐citrulline MMAE drug linker. MMAE can then exert a bystander effect on adjacent tumor cells regardless of their HER2 status [20]. RC48 has shown promising antitumor activity in preclinical models of breast [18], gastric [21], and ovarian cancers [22] with different levels of HER2 expression.

Currently, neoadjuvant therapy for TNBC primarily involves anthracycline‐based regimens administered concurrently with or sequentially before taxanes, with a pCR rate of approximately 40% [23]. Previous studies such as KEYNOTE‐522 and IMpassion031 have shown that adding immune checkpoint inhibitors to anthracycline‐based neoadjuvant chemotherapy improves pCR in early‐stage TNBC, increasing pCR from 51.2 to 64.8% in KEYNOTE‐522 and from 41 to 58% in IMpassion031 [11, 12]. Based on this, major guidelines both domestically and internationally recommend immunotherapy combined with chemotherapy for neoadjuvant treatment of TNBC. However, due to the severe cardiac toxicity caused by anthracyclines, there is growing interest in anthracycline‐free regimens. Penpulimab is a novel humanized anti‐programmed death 1 (anti‐PD‐1) IgG1 monoclonal antibody with a modified Fc fragment structure that completely eliminates antibody‐dependent cellular cytotoxicity (ADCC)/antibody‐dependent cellular phagocytosis (ADCP)/complement‐dependent cytotoxicity (CDC) effects to avoid effector T cell depletion. Additionally, the Fc fragment modification reduces antibody‐dependent cytokine release effects, decreases IL‐8 and IL‐6 release, thereby further enhancing immune efficacy and reducing immune‐related adverse events (irAEs).

RC48 and immune checkpoint inhibitors have demonstrated independent antitumor activity, and several complementary mechanisms provide a strong biological rationale for combining them in HER2‐low breast cancer. First, ADCs carrying microtubule‐targeting payloads such as MMAE, the cytotoxic moiety of RC48, can trigger immunogenic forms of tumor cell death. This process is accompanied by extracellular release of high‐mobility group box 1 (HMGB1), translocation of calreticulin to the cell surface, and active secretion of adenosine triphosphate (ATP), all of which serve as damage‐associated molecular patterns that facilitate dendritic cell (DC) recruitment, antigen uptake, and subsequent cross‐priming of tumor‐specific cluster of differentiation 8‐positive (CD8^+^) T cells [24, 25]. Second, the resulting wave of tumor cell killing in situ liberates a broader repertoire of tumor antigens and is typically followed by enhanced lymphocyte trafficking into the tumor bed, a shift that effectively reshapes a poorly inflamed (“cold”) microenvironment into one in which programmed cell death protein 1 (PD‐1)/PD‐L1blockade is more likely to be effective [26]. Third, this conceptual framework is supported by experimental work specific to the RC48–anti‐PD‐1 pairing: in HER2‐transgenic mouse models, the combination achieved complete tumor regression and conferred protective immunological memory upon rechallenge, neither of which was reproduced by monotherapy with either agent [27]. Fourth, penpulimab is an Fc‐engineered humanized anti‐PD‐1 IgG1 antibody whose constant region has been modified to abolish ADCC, ADCP, and CDC activity. This design preserves the effector T‐cell populations being recruited and reactivated by ADC‐induced immunogenic cell death, and is therefore particularly well suited as a checkpoint‐blockade partner for an ADC‐based regimen. However, its potential in the neoadjuvant setting for HER2‐low breast cancer remains largely unexplored.

On this basis, we conducted an exploratory single‐arm clinical study of RC48 plus penpulimab in patients with HER2‐low early or locally advanced breast cancer, with the primary objective of assessing efficacy and safety. More importantly, multiomic profiling—including proteomic, transcriptomic, and immune‐cell analyses—was integrated to explore candidate biomarkers of response and to generate hypotheses for future biomarker‐driven validation studies in this neoadjuvant setting.

## 2 Results

### 2.1 Patient Disposition and Baseline Characteristics

From August 14, 2023 to August 7, 2024, 50 patients were screened and 20 met eligibility and initiated neoadjuvant therapy; these 20 patients constituted both the intention‐to‐treat (ITT) and safety populations (Figure 1A). Among the 30 patients who failed screening, the main reasons for exclusion were thyroiditis, staging that did not meet the eligibility criteria, and patient refusal to participate. Enrolled patients received RC48 plus penpulimab every 3 weeks (RC48 2.0 mg/kg IV; penpulimab 200 mg IV) for up to six cycles (Figure 1B). Sixteen patients (80%) completed all planned neoadjuvant cycles and proceeded to surgery. Four patients (20%) were not evaluable for the primary end point: one discontinued because of an adverse event, one withdrew consent at the patient's request, and two discontinued for other reasons (disease progression and per investigator discretion) (Figure 1A). Serial biospecimen collection was performed as prespecified: tumor tissue at baseline before neoadjuvant therapy (pre‐NAT) and at surgery after neoadjuvant therapy (post‐NAT); and peripheral blood at baseline, cycle 3 Day 1 (C3D1), cycle 5 Day 1 (C5D1), and surgery. Tissue specimens were used for pathology and tissue‐based assays, including IHC, multiplex immunofluorescence (mIF), and bulk RNA sequencing (RNA‐seq) where applicable, whereas plasma/serum specimens were used for Olink proteomics (Figure 1B).

**FIGURE 1:**
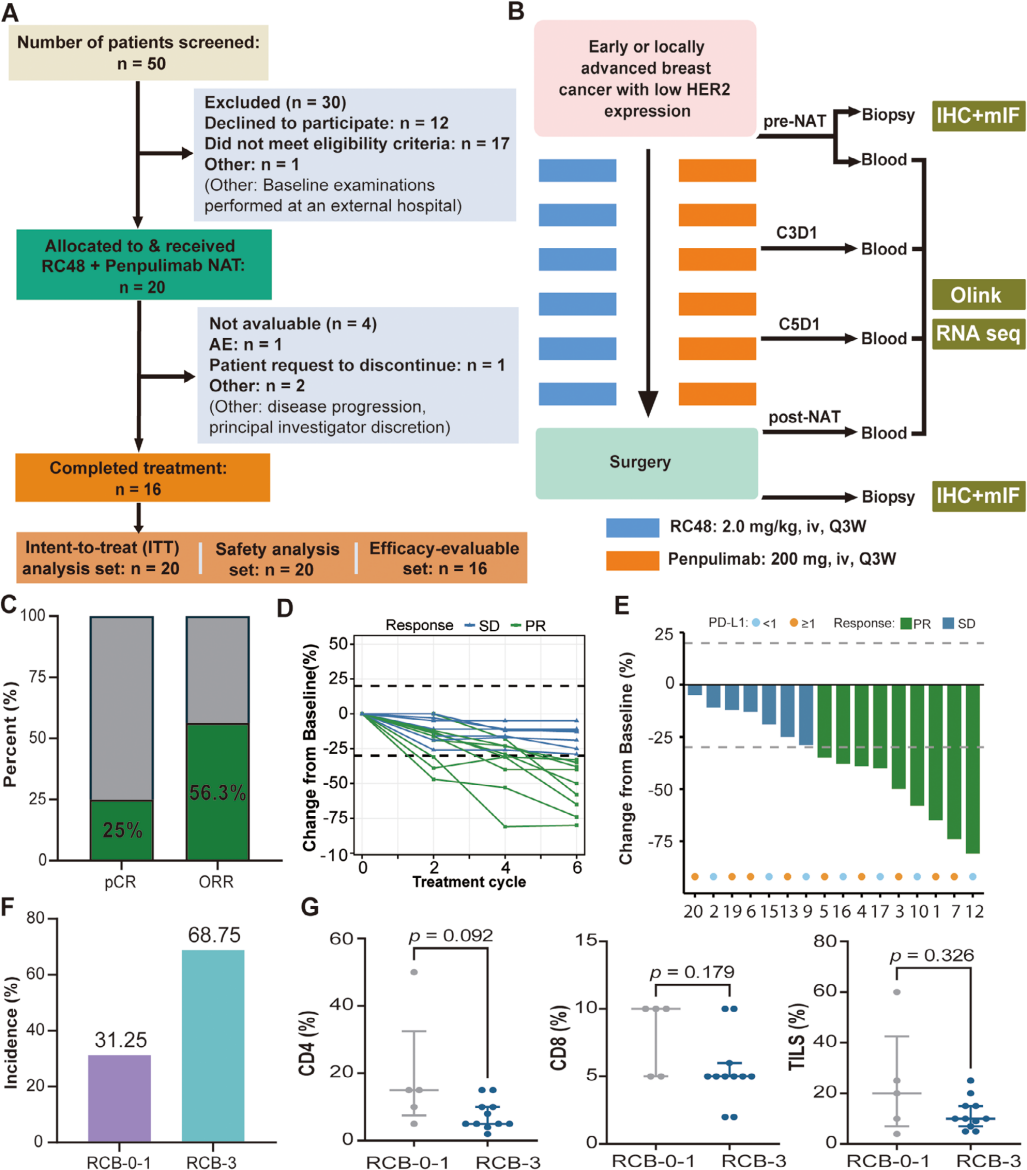
Study design, patient disposition, and efficacy outcomes. (A) Patient disposition. Of 50 screened patients, 30 were excluded and 20 were enrolled and received RC48 plus penpulimab neoadjuvant therapy; 16 completed treatment and four discontinued (adverse event, *n* = 1; withdrawal, *n* = 1; other reasons, *n* = 2). The ITT, safety, and efficacy‐evaluable populations comprised 20, 20, and 16 patients, respectively. (B) Study schema showing treatment and sample collection at baseline, cycle 3 Day 1 (C3D1), cycle 5 Day 1 (C5D1), and posttreatment surgery. (C) In the evaluable cohort (*n* = 16), the pathologic complete response (pCR; ypT0/is, ypN0) rate was 25% (four out of 16) and the objective response rate (ORR) was 56.3% (nine out of 16). (D) Spider plot of tumor size change over time in the evaluable cohort (*n* = 16); dashed lines indicate RECIST thresholds. (E) Waterfall plot of best percentage change from baseline in the evaluable cohort (*n* = 16), color‐coded by response and annotated with PD‐L1 status. (F) Residual cancer burden (RCB) distribution at surgery (*n* = 16; RCB 0–1, 31.25%, *n* = 5; RCB‐3, 68.75%, *n* = 11). (G) Immune infiltration by RCB class (*n* = 16; RCB 0–1, *n* = 5; RCB‐3, *n* = 11); no significant differences were observed in CD4^+^, CD8^+^, or total TILs. AE, adverse event; C3D1/C5D1, cycle 3/5 Day 1; IHC, immunohistochemistry; IV, intravenous; mIF, multiplex immunofluorescence; NAT, neoadjuvant therapy; ORR, objective response rate; pCR, pathologic complete response; PD, progressive disease; PR, partial response; RC48, disitamab vedotin; RCB, residual cancer burden; RECIST, response evaluation criteria in solid tumors; SD, stable disease.

Baseline characteristics of the ITT population (*N* = 20) are summarized in Table 1. The median age was 43.5 years (range, 33–68), and all patients were female with Eastern Cooperative Oncology Group (ECOG) performance status of 0. Half of the tumors were HER2 IHC 1^+^ and half were IHC 2^+^/FISH^−^. Most patients had high proliferative disease, with Ki‐67>20% in 80%. HR positivity was common (ER 90%, PR 85%). The majority presented with T2 (65%) and node‐positive (85%) disease, with 65% at Stage II and 35% at Stage III. PD‐L1 CPS was ≥1 in 55% of patients.

**TABLE 1:** Baseline demographic and disease characteristics (ITT population, *N* = 20).

Patient characteristic	All ITT patients
Median age, years	43.5 (33,68)
Female, *n* (%)	20 (100)
ECOG‐PS, *n* (%)	
0	20 (100)
1	0
HER2, *n* (%)	
IHC 1^+^	10 (50.0)
IHC 2^+^/FISH^−^	10 (50.0)
Ki‐67 score, *n* (%)	
≤20%	4 (20.0)
>20%	16 (80.0)
HR, *n* (%)	
ER^+^	18 (90.0)
PR^+^	17 (85.0)
T stage, *n* (%)	
T1	2 (10.0)
T2	13 (65.0)
T3	1 (5.0)
T4	4 (20.0)
N stage, *n* (%)	
N0	3 (15.0)
N1	15 (75.0)
N2	2 (10.0)
N3	0
Stage (cTNM classification), *n* (%)	
II	13 (65.0)
III	7 (35.0)
PD‐L1, *n* (%)	
CPS ≥1	11 (55.0)
CPS <1	9 (45.0)

Data are *n* (%) unless otherwise indicated. Age is median (range).

Abbreviations: CPS, combined positive score; cTNM, clinical tumor–node–metastasis; ECOG PS, Eastern Cooperative Oncology Group performance status; ER, estrogen receptor; FISH, fluorescence in situ hybridization; HER2, human epidermal growth factor receptor 2; HR, hormone receptor; IHC, immunohistochemistry; PD‐L1, programmed death‐ligand 1; PR, progesterone receptor.

### 2.2 Efficacy and Safety

In the ITT population (*N* = 20), the pCR (ypT0/is, ypN0) rate was 20.0% (four out of 20; 95% CI 5.7–43.7%) and the objective response rate (ORR) was 45.0% (nine out of 20; 95% CI 23.1–68.5%). In the per‐protocol (PP) set (*N* = 16, baseline characteristics are summarized in Table 2), pCR was 25.0% (four out of 16; 95% CI 7.3–52.4%) and ORR 56.3% (nine out of 16; 95% CI 29.9–80.2%) (Figure 1C). Given the limited cohort size, these efficacy estimates were descriptive and should be interpreted with caution. In particular, the wide exact 95% confidence interval for pCR in the PP population reflects substantial statistical uncertainty and limits the ability to draw definitive conclusions regarding treatment efficacy. Longitudinal assessments showed that, except for progressive disease (PD) patients, most patients experienced early and sustained tumor shrinkage, with maximum reductions approaching 75% (Figure 1D, data of PD patients are shown in Figure S1A). Waterfall plots confirmed substantial reductions in tumor burden across the cohort, and responses were observed irrespective of PD‐L1 expression levels (Figure 1E, data for the ITT population are shown in Figure S1B). At surgery, 31.25% of patients achieved residual cancer burden (RCB) 0–1, while 68.75% were classified as RCB‐3 (Figure 1F). However, no significant differences in cluster of differentiation 4‐positive (CD4^+^), CD8^+^, or tumor‐infiltrating lymphocytes (TILs) percentages were observed between RCB 0–1 and RCB‐3 groups (Figure 1G). Exploratory subgroup analysis (Figure S1C) showed numerically variable pCR rates across clinical subgroups: 22.2% in Stage II and 28.6% in Stage III disease, 28.6% among lymph node (LN)‐positive and 0% among LN‐negative patients, 14.3% for PD‐L1 CPS <1 and 75.0% for PD‐L1 CPS ≥10, 50.0% in ER^+^<10% and 21.4% in ER^+^ ≥10%, and 37.5% in HER2 IHC 2^+^/FISH^−^ versus 12.5% in HER2 IHC 1^+^ tumors. Given the small number of patients in each subgroup, these findings were descriptive only and may reflect baseline imbalance or confounding rather than true predictive effects.

**TABLE 2:** Baseline demographic and disease characteristics (evaluable population, *N* = 16).

Pathological complete response rate	Evaluable patients *n* (%)
Pathological complete response rate (tpCR) (ypT0/is ypN0)	4 (25)
Stage (cTNM classification)	
Stage II	9 (56.3)
Stage III	7 (43.7)
Nodal status	
Lymph node involvement‐positive	14 (87.5)
Lymph node involvement‐negative	2 (12.5)
PD‐L1	
PD‐L1 CPS < 1	7 (43.8)
PD‐L1 CPS ≥ 10	3 (18.8)
ER	
ER^+^ < 10%	2 (12.5)
ER^+^ ≥ 10%	14 (87.5)
ER^+^ ≥ 10% & PD‐L1 CPS ≥ 10	3 (18.8)
HER2	
HER2 IHC 2^+^ /FISH^−^	8 (50)
HER2 IHC 1^+^	8 (50)
Ki‐67 scores	
>20%	12 (75.0)
≤20%	4 (25.0)

*Note*: PD‐L1 CPS distribution among the 16 evaluable patients was as follows: CPS < 1, 7 (43.8%); 1 ≤ CPS < 10, 6 (37.5%); and CPS ≥ 10, 3 (18.8%). Only the CPS < 1 and CPS ≥ 10 threshold groups are displayed in the table.

Abbreviations: CPS, combined positive score; cTNM, clinical tumor–node–metastasis; ER, estrogen receptor; FISH, fluorescence in situ hybridization; HER2, human epidermal growth factor receptor 2; IHC, immunohistochemistry; PD‐L1, programmed death‐ligand 1.

Given the predominance of HR‐positive disease in this cohort and the limited ability of pCR alone to capture long‐term risk in this subtype, we further evaluated paired Ki‐67 dynamics and detailed RCB. Paired baseline and posttreatment Ki‐67 measurements were available for 16 patients in the evaluable population; 14 had ER expression ≥10%. Across the evaluable cohort, median Ki‐67 declined from 25% at baseline to 15% at surgery, with a median per‐patient reduction of 2.5 percentage points; the corresponding values among HR‐positive patients were 25, 17.5, and 2.5 percentage points.

Detailed RCB assessment was also performed. In the evaluable population, the median continuous RCB score was 3.390. RCB‐0, RCB‐I, RCB‐II, and RCB‐III were observed in 4 (25.0%), 1 (6.3%), 0, and 11 (68.8%) patients, respectively. All 16 evaluable patients were HR‐positive; therefore, the median RCB score and the corresponding RCB‐class distribution in the evaluable HR‐positive population were identical. Together, these analyses characterize residual proliferative activity and disease burden beyond what pCR status alone conveys.

To improve clinical interpretability, we additionally summarized efficacy outcomes according to clinical subtype. HR‐positive/HER2‐low disease was defined as ER‐positive and/or PR‐positive with HER2‐low status, whereas TNBC/HER2‐low disease was defined as ER‐negative, PR‐negative, and HER2‐low. In the ITT population, 18 patients (90.0%) had HR‐positive/HER2‐low disease and two patients (10.0%) had TNBC/HER2‐low disease. Both TNBC/HER2‐low patients were not evaluable for the primary efficacy analysis, leaving 16 HR‐positive/HER2‐low patients in the evaluable population. Among HR‐positive/HER2‐low patients, the pCR rate was 25.0% (four out of 16) in the evaluable population and 22.2% (four out of 18) in the ITT population with nonresponder imputation; the corresponding ORRs were 56.3% (nine out of 16) and 50.0% (nine out of 18), respectively. Because no TNBC/HER2‐low patients were evaluable, subtype‐specific efficacy in this subgroup could not be assessed. These analyses are descriptive and intended to improve clinical interpretability; no formal statistical comparison between subtypes was performed given the very limited TNBC/HER2‐low sample size.

Safety outcomes were assessed in the ITT population (*N* = 20; Table 3). All 20 patients (100%) experienced at least one treatment‐emergent adverse event (TEAE), and five patients (25.0%) experienced Grade ≥3 TEAEs. Overall, five patients (25.0%) required adverse event‐related treatment modification, comprising treatment delay and permanent treatment discontinuation. Specifically, treatment delay occurred in four patients (20.0%) and permanent treatment discontinuation occurred in one patient (5.0%). The main adverse events leading to treatment modification were hepatic dysfunction (15.0%) and autoimmune myocarditis (5.0%). Hematologic events were mostly low grade: neutropenia 4 (20%), leukopenia 3 (15%), and anemia 3 (15%) (all Grade 1–2). Common laboratory/metabolic abnormalities included alanine aminotransferase (ALT) increase 13 (65%; Grade ≥3, 1 [5%]), aspartate aminotransferase (AST) increase 10 (50%; Grade ≥3, 1 [5%]), hypertriglyceridemia 10 (50%; Grade ≥3, 1 [5%]), and hyperglycemia 6 (30%). Frequent gastrointestinal/dermatologic events were constipation 12 (60%), nausea 11 (55%), rash 11 (55%), pruritus 14 (70%), diarrhea 4 (20%), and vomiting 3 (15%). Other TEAEs included urinary tract infection 7 (35%; Grade ≥3, 1 [5%]), fatigue 8 (40%), pain 8 (40%), anorexia 5 (25%), paresthesia 8 (40%), and abnormal urinalysis 5 (25%; Grade ≥3, 1 [5%]).

**TABLE 3:** Treatment‐emergent adverse events (ITT population, *N* = 20).

	*N* = 20		
AE	Any grade *n* (%)	Grade 1–2 *n* (%)	≥Grade 3 *n* (%)
Treatment‐emergent adverse events (TEAEs)	20 (100)	15 (75.0)	5 (25.0)
Treatment discontinuation or delay due to adverse events (AEs)	5 (25.0)	4 (20.0)	1 (5.0)
Leukopenia	3 (15.0)	3 (15.0)	0
Neutropenia	4 (20.0)	4 (20.0)	0
Anemia	3 (15.0)	3 (15.0)	0
ALT increased	13 (65.0)	12 (60.0)	1 (5.0)
AST increased	10 (50.0)	9 (45.0)	1 (5.0)
Hyperglycemia	6 (30.0)	6 (30.0)	0
Elevated triglycerides	10 (50.0)	9 (45.0)	1 (5.0)
Abnormal urinalysis	5 (25.0)	4 (20.0)	1 (5.0)
Nausea	11 (55.0)	11 (55.0)	0
Vomiting	3 (15.0)	3 (15.0)	0
Diarrhea	4 (20.0)	4 (20.0)	0
Constipation	12 (60.0)	12 (60.0)	0
Rash	11 (55.0)	11 (55.0)	0
Pruritus	14 (70.0)	14 (70.0)	0
Pain	8 (40.0)	8 (40.0)	0
Anorexia	5 (25.0)	5 (25.0)	0
Fatigue	8 (40.0)	8 (40.0)	0
Paresthesia	8 (40.0)	8 (40.0)	0
Urinary tract infection	7 (35.0)	6 (30.0)	1 (5.0)
**ADC related to adverse events**			
Alopecia	18 (90.0)	18 (90.0)	0
Insomnia	5 (25.0)	5 (25.0)	0
Paresthesia	8 (40.0)	8 (40.0)	0
Allergic dermatitis	1 (5.0)	1 (5.0)	0
**Immune‐related to adverse events**			
Hypothyroidism	4 (20.0)	4 (20.0)	0
Immune‐mediated myocarditis	1 (5.0)	0	1 (5.0)
Hyperthyroidism	3 (15.0)	2 (10.0)	1 (5.0)
Urticaria	1 (5.0)	1 (5.0)	0
Proteinuria	1 (5.0)	1 (5.0)	0

Events are summarized by worst grade (any grade, Grade 1–2, and Grade ≥3).

Abbreviations: ADC, antibody–drug conjugate; CTCAE, Common Terminology Criteria for Adverse Events; irAE, immune‐related adverse event; TEAE, treatment‐emergent adverse event.

Adverse events considered ADC‐related were frequent, led by alopecia 18 (90%), with paresthesia 8 (40%), insomnia 5 (25%), and allergic dermatitis 1 (5%). Prespecified immune‐related AEs included hypothyroidism 4 (20%), hyperthyroidism 3 (15%; Grade ≥3, 1 [5%]), immune‐mediated myocarditis 1 (5%; Grade 3), urticaria 1 (5%), and proteinuria 1 (5%). One patient developed Grade 3 immune‐mediated myocarditis during neoadjuvant therapy. In July 2024, after two cycles of treatment, electrocardiography revealed sinus tachycardia, premature ventricular contractions, and ST‐segment changes (coved ST‐segment elevation in leads V1–V5). Cardiac biomarkers were markedly elevated, with myoglobin of 541.00 ng/mL, creatine kinase–MB (mass) of 116.00 ng/mL, and troponin of 354.0 ng/L. The event was managed with immediate treatment interruption, cardiology consultation, and cardiac monitoring, together with systemic corticosteroid therapy: intravenous methylprednisolone was initiated in the emergency department and, following clinical stabilization, transitioned to an oral regimen. Cardiac biomarkers were monitored weekly, and the corticosteroid dose was tapered by 4 mg at each step in accordance with the patient's biochemical response. Cardiac biomarkers returned to normal values by October 2024, and no myocarditis‐related death occurred.

Overall, toxicities associated with the regimen were largely confined to low‐grade events, with high‐grade occurrences being sporadic and comprising primarily laboratory abnormalities and isolated immune‐mediated myocarditis.

### 2.3 Baseline Correlates of Pathologic Response

To explore baseline determinants of response, we compared pretreatment profiles between patients who achieved pCR and those who did not, using Olink proteomics, mIF, IHC, and RNA sequencing.

Seven proteins (IL13, CSF‐1, uPA, TNFRSF9, β‐NGF, CCL19, and CCL11) were significantly upregulated in the pCR group (Figures 2A–C and S2), most of which are involved in immune cell recruitment and polarization. Kyoto Encyclopedia of Genes and Genomes (KEGG) analysis of these proteins showed that they were significantly enriched in cytokine–cytokine receptor interaction, IL‐17 signaling pathway, NF‐κB signaling pathway, and chemokine signaling pathway (Figure 2D), suggesting that the tumor microenvironment (TME) in the pCR group showed a greater immune cell recruitment ability and proinflammatory signaling activity.

**FIGURE 2:**
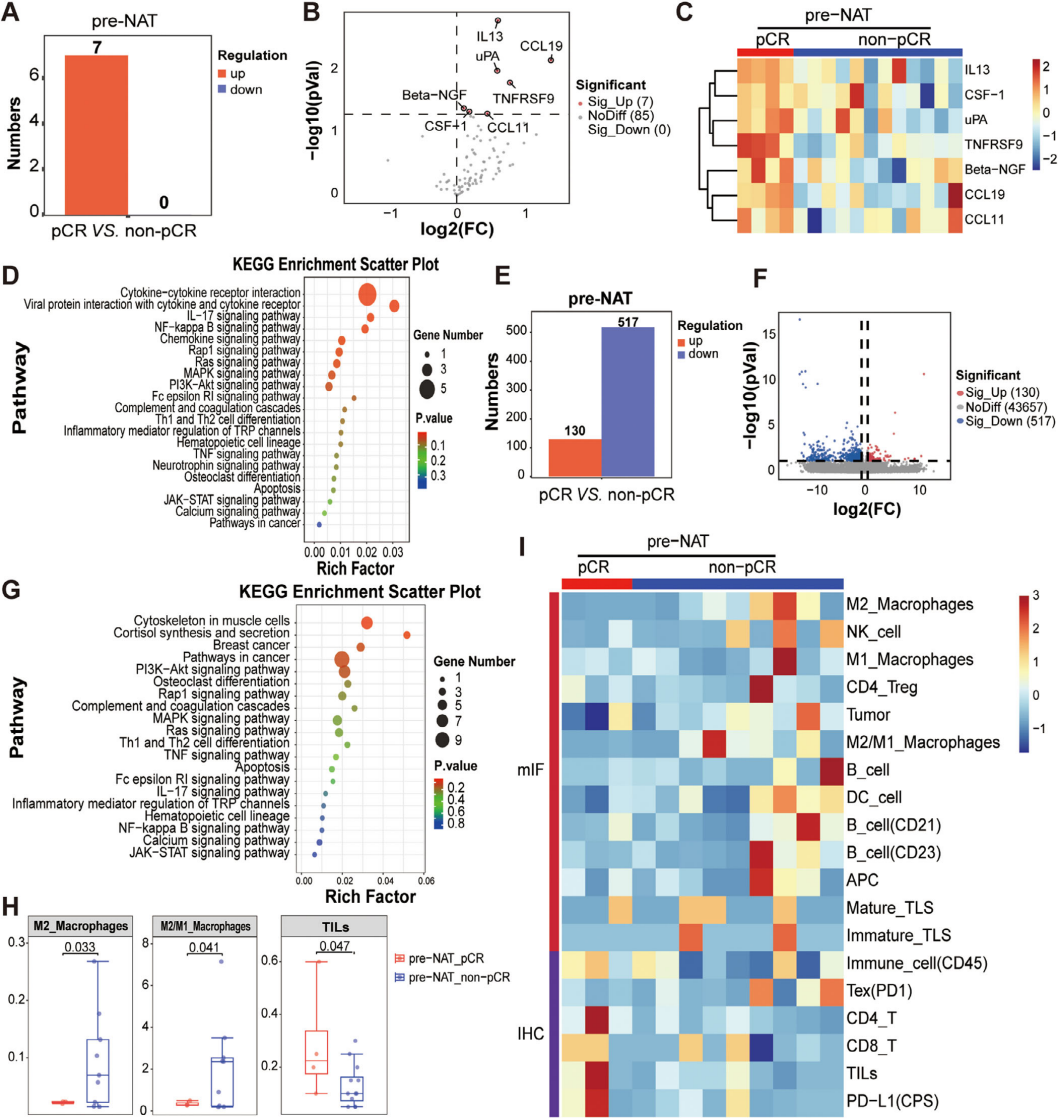
Baseline proteomic, immune‐cell, and transcriptomic features associated with pCR. (A) Number of differentially expressed proteins between pCR (*n* = 4) and non‐pCR (*n* = 12) tumors at baseline by Olink analysis. (B) Volcano plot showing significantly upregulated proteins in the pCR group (*n* = 4) versus non‐pCR group (*n* = 12) (IL13, CSF‐1, uPA, TNFRSF9, β‐NGF, CCL19, CCL11). (C) Heatmap of the seven proteins across individual patients (pCR, *n* = 4; non‐pCR, *n* = 12), and (D) KEGG analysis of these proteins. (E) Number of DEGs between baseline pCR (*n* = 4) and non‐pCR (*n* = 10) tumors. (F) Volcano plot of DEGs and (G) KEGG analysis of these DEGs (pCR, *n* = 4; non‐pCR, *n* = 10). (H) Baseline immune profiling by mIF and IHC (pCR, *n* = 3; non‐pCR, *n* = 9). (I) Boxplots showing increased M2 macrophages and M2/M1 ratios in non‐pCR tumors (*n* = 9) and higher TILs density in pCR tumors (*n* = 3). DEG, differentially expressed gene; IHC, immunohistochemistry; KEGG, Kyoto Encyclopedia of Genes and Genomes; mIF, multiplex immunofluorescence; NAT, neoadjuvant therapy; pCR, pathologic complete response.

RNA‐seq revealed extensive transcriptional differences between the pretreatment pCR and non‐pCR groups (Figure S3). Several key immune‐related genes were significantly upregulated in the pCR group (Figure 2E,F), such as T‐cell‐receptor‐related genes TRBV6‐5 and TRAV29DV5, B‐cell‐receptor‐related genes IGHV4‐39 and IGLC1, and immune regulatory genes CD1E and CD200R1. KEGG enrichment analysis further revealed that these DEGs were significantly enriched in multiple signaling pathways (Figure 2G), among which immune‐related pathways (such as IL‐17, TNF, NF‐κB, JAK–STAT, hematopoietic cell lineage, Th1/Th2 differentiation, etc.) were enriched. The results suggested a trend toward stronger immune‐cell activation and recruitment signals in the TME of pCR group. These transcriptional patterns were broadly concordant with the proteomic findings, suggesting that tumors achieving pCR may have had a more immune‐active baseline microenvironment.

Building on these molecular findings, we examined the corresponding immune‐cell phenotypes spatially using mIF combined with pan‐CK‐based tumor/stroma segmentation. The non‐pCR group showed higher M2 macrophage infiltration level (Figure 2H,I; *p *= 0.033) and M2/M1 macrophage ratio (Figure 2H,I; *p *= 0.041), suggesting that the TME in this group had a more immunosuppressive phenotype. In contrast, the pCR group contained more TILs (Figure 2H,I; *p *= 0.047), which was consistent with the upregulation of lymphocyte chemokines (such as CCL19) in the proteome and the overall active trend of chemotactic signals in the transcriptome. Although other immune cells (such as CD8^+^ T cells and CD4^+^ T cells) did not reach statistical significance, the spatial distribution trend was consistent with the above findings (Figure S4).

In summary, integrated multiomics and spatial data suggested that pCR‐associated tumors may have exhibited a more immune‐active baseline microenvironment, whereas non‐pCR tumors showed features consistent with macrophage‐associated immunosuppression.

### 2.4 Exploratory Development and Internal Assessment of a Candidate Baseline Biomarker Score

To identify underlying baseline correlates of response, we integrated proteomic, immune‐cell, and transcriptomic features. Immune composition inferred by xCell showed distinct patterns between groups (Figure 3A), with pCR tumors enriched for CD4^+^ naive T cells (Figure 3B; *p *= 0.042) and CD8^+^ effector memory T cells (Figure 3C; *p *= 0.012). Gene set enrichment analysis (GSEA) demonstrated preferential activation of chemokine and TNF/NF‐κB signaling in pCR tumors (Figure 3D). Proteomic and transcriptomic analyses both revealed concordant pathway trends, with significance predominantly observed in the proteome, particularly in cytokine–cytokine receptor interaction, IL‐17, NF‐κB, and chemokine signaling pathways (Figure 3E).

**FIGURE 3:**
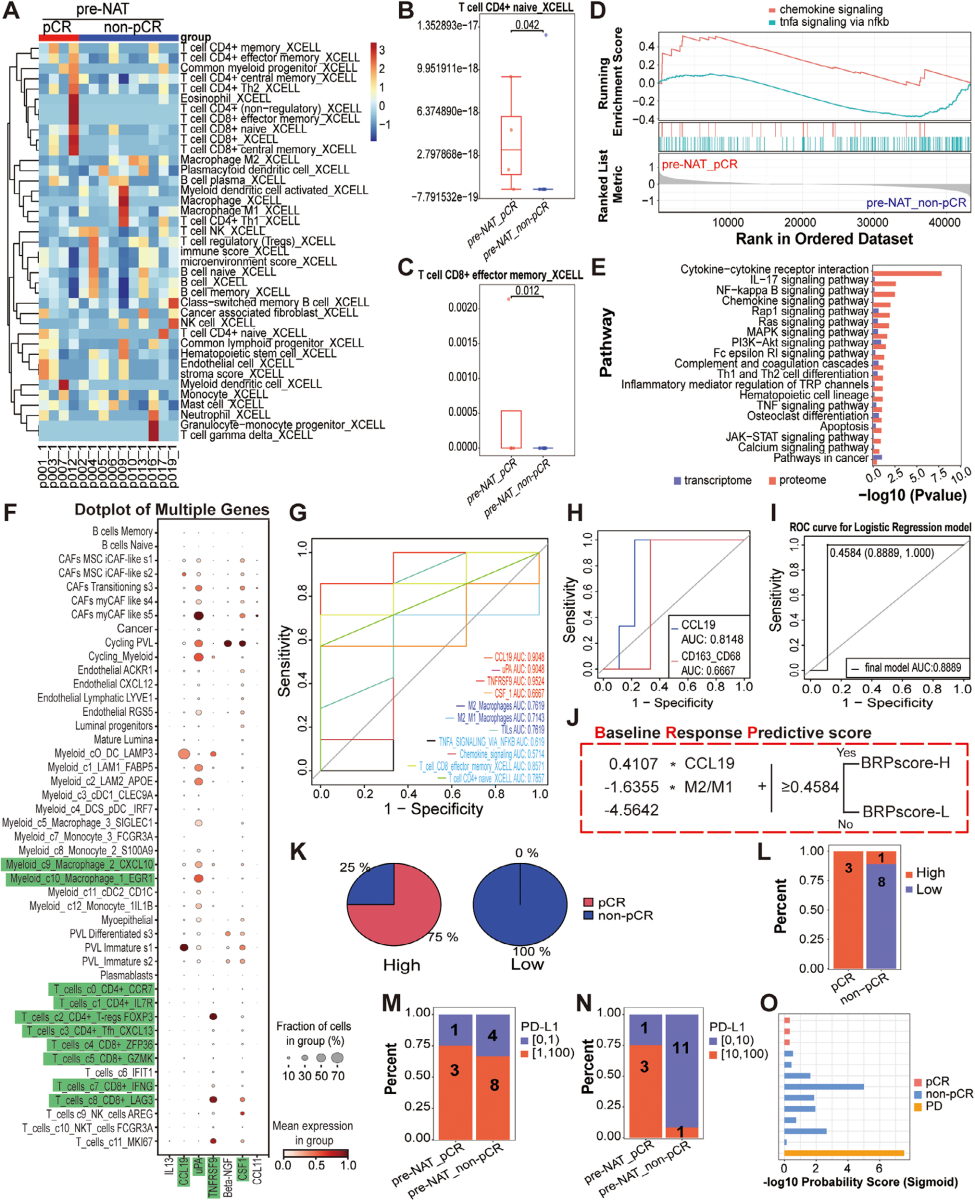
Baseline integrative analysis and exploratory BRPscore assessment. (A) xCell deconvolution of baseline RNA‐seq revealed distinct immune‐cell patterns between pCR (*n* = 4) and non‐pCR tumors (*n* = 10). (B and C) Boxplots of selected immune subsets showing enrichment of CD4^+^ naive T cells and CD8^+^ effector memory T cells in pCR tumors (pCR, *n* = 4; non‐pCR, *n* = 10). (D) GSEA indicated preferential activation of chemokine and TNF/NF‐κB signaling in pCR tumors (pCR, *n* = 4; non‐pCR, *n* = 10). (E) KEGG pathway analysis of proteome‐ and transcriptome‐derived features showed significance predominantly in the proteome (pCR, *n* = 4; non‐pCR, *n* = 10). (F) Protein–cell mapping identified four (TNFRSF9, uPA, CSF‐1, and CCL19) of seven pCR‐associated proteins as immune‐relevant biomarkers. (G) ROC screening of prioritized proteins, immune‐cell subsets, and pathways. (H–I) Exploratory two‐variable logistic model incorporating CCL19 and the M2/M1 ratio, showing an apparent AUC of 0.8889 before internal validation. (J) Formula of the exploratory Baseline Response Predictive score (BRPscore) derived from these two variables. (K–L) Descriptive stratification by BRPscore High versus Low. *N* = 12 (pCR, *n* = 3; non‐pCR, *n* = 9), representing patients who completed NAT and had available Olink proteomics and multiplex immunofluorescence data. (M and N) Descriptive comparison with PD‐L1 CPS thresholds of 1 and 10 (pCR, *n* = 4; non‐pCR, *n* = 12). (O) Per‐patient predicted probabilities shown for exploratory visualization. *N* = 13 (pCR, *n* = 4; non‐pCR, *n* = 8; PD, *n* = 1), representing patients with available Olink proteomics and mIF data. AUC, area under the ROC curve; BRPscore, Baseline Response Predictive score; GSEA, gene set enrichment analysis; KEGG, Kyoto Encyclopedia of Genes and Genomes; mIF, multiplex immunofluorescence; NAT, neoadjuvant therapy; pCR, pathologic complete response; PD, progressive disease; ROC, receiver operating characteristic.

Then, we mapped the previously identified seven pCR‐enriched proteins (Figure 2B) to cell types using reference single‐cell datasets and four proteins (Figure 3F)—CCL19, uPA, TNFRSF9, and CSF‐1—were prioritized based on their immune relevance and receptor–ligand connectivity, providing a focused panel of candidate biomarkers for further analysis.

To integrate baseline protein, immune‐cell, and pathway features, we performed multivariable logistic regression using the four prioritized proteins together with identified immune‐cell subsets and pathways (Figure 3G). In this small biomarker‐evaluable subset, neither CCL19 (*β* = 0.4107, standard error = 0.6168, *p *= 0.505) nor the M2/M1 macrophage ratio (*β* = −1.6355, standard error = 1.8896, *p *= 0.387) reached statistical significance in the regression model, a finding likely attributable to the limited number of patients and pCR events. The apparent area under the receiver operating characteristic curve (AUC) of the exploratory Baseline Response Predictive score (BRPscore) was 0.8889, but this estimate should be interpreted cautiously because it was derived and tested in the same dataset (Figure 3H,I). Based on the coefficients obtained from univariate analysis or prior knowledge, we further constructed the BRPscore: logit(p = NAT_pCR) = 0.4107*CCL19‐1.6355*M2/M1‐4.5642. For exploratory baseline stratification, we constructed a BRPscore by combining baseline CCL19 level and the M2/M1 macrophage ratio in a logistic regression model for pCR prediction. The cutoff was determined by maximizing the Youden index on the apparent receiver operating characteristic (ROC) curve. The optimal cutoff value was 0.4584, with BRPscore ≥0.4584 used to define the predicted pCR subgroup (Figure 3J).

Using the exploratory BRPscore threshold, patients were stratified into BRPscore‐High and ‐Low groups. In this small biomarker‐evaluable subset, pCR cases with available matched data were observed in the BRPscore‐High group, whereas most non‐pCR cases were observed in the BRPscore‐Low group (Figure 3K–L). Although the BRPscore showed apparent discrimination between pCR and non‐pCR cases in the development dataset, with an apparent AUC of 0.8889, its performance decreased after internal validation. Internal validation using leave‐one‐out cross‐validation (LOOCV) yielded a markedly lower AUC of 0.6667 (95% CI: 0.3333–0.8889). Bootstrap optimism correction (10,000 iterations) gave an optimism‐corrected AUC of 0.8375, while the bootstrap resample apparent AUC showed a very wide 95% confidence interval (0.5625–1.0000). Therefore, this BRPscore should be interpreted as an exploratory baseline biomarker signal rather than a clinically deployable classifier, and both the cutoff and predictive performance require validation in larger, independent cohorts.

As a benchmark, we applied two commonly used PD‐L1 thresholds (1 and 10). At the 1 cut‐off, there remained substantial overlap between pCR and non‐pCR, with limited discriminatory ability (Figure 3M). At the CPS 10 threshold, descriptive separation appeared greater than at CPS 1, but overlap remained and the number of patients was too small for formal comparison (Figure 3N). Per‐patient predicted probabilities are shown descriptively in Figure 3O. The PD case showed a low predicted probability of pCR—consistent with the exploratory direction of the score—but this observation should not be interpreted as evidence of model reliability given the limited sample size.

### 2.5 Rapid Phase Molecular Dynamics

To further explore the biological basis of treatment response, we first compared the C3D1 proteome with baseline to capture the rapid response phase. C3D1 was selected as the early on‐treatment biomarker time point because it allowed sufficient time (two full cycles, ∼6 weeks) for ADC‐ and PD‐1‐blockade‐related proteomic changes to emerge, aligned with the reported 4–6 week peak window for cytokine/immune‐checkpoint modulation in ADC + ICI combinations, and immediately preceded the mid‐treatment imaging assessment, enabling prospective response correlation.

In the pCR group, 10 proteins increased and two decreased, whereas in the non‐pCR group, 33 proteins increased and none decreased significantly (Figure 4A–C). Venn analysis (Figure 4D) identified five proteins uniquely altered in pCR (IL‐22RA1, 4E‐BP1, CD6, MCP‐1, and MCP‐3), 26 uniquely altered in non‐pCR, and seven shared between groups. Among the non‐pCR‐specific proteins, seven proteins (Beta‐NGF, IL‐10RB, IL‐17C, IL‐4, NT‐3, OPG, and uPA) exhibited the opposite direction of change in pCR. Within the shared set of seven proteins, only SLAMF1 changed in opposite directions between pCR and non‐pCR. In total, 13 proteins were identified as potential signatures, including five uniquely altered in pCR, seven in non‐pCR, and one shared protein with divergent changes between groups. Among the 13 proteins, MCP‐1 and IL4 are shown as examples (Figure 4E,F). KEGG analysis of those 13 proteins suggested enrichment patterns in cytokine/cytokine–receptor interaction, PI3K–AKT, and NF‐κB/TNF‐related signaling (Figure 4G).

**FIGURE 4:**
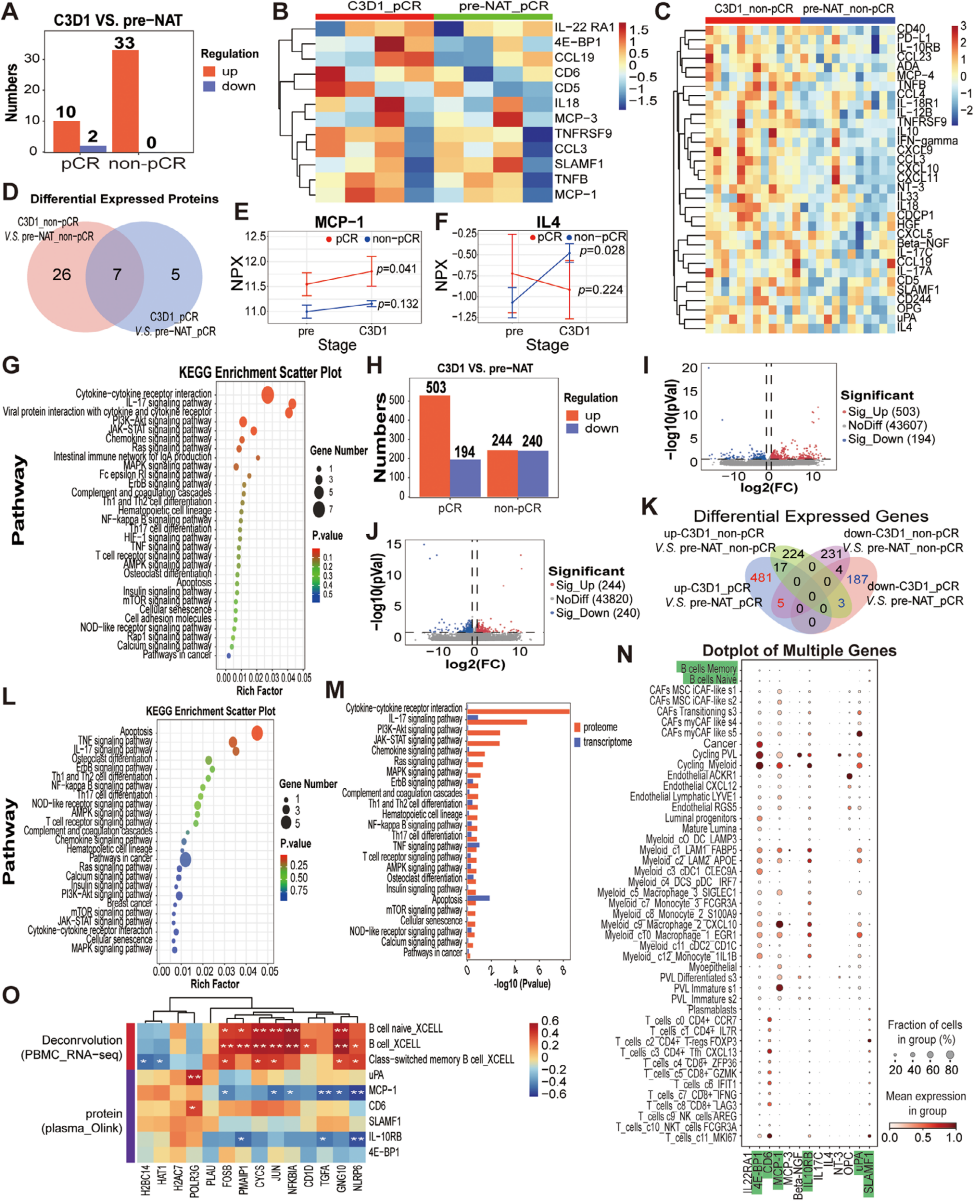
Proteomic and transcriptomic dynamics during the rapid response phase (C3D1). (A) Numbers of proteins significantly altered at C3D1 relative to baseline in pCR (pre‐NAT_pCR, *n* = 4; C3D1_pCR, *n* = 4) and non‐pCR (pre‐NAT_non‐pCR, *n* = 12; C3D1_non‐pCR, *n* = 12) tumors. (B and C) Heatmaps of differentially expressed proteins in pCR (pre‐NAT_pCR, *n* = 4; C3D1_pCR, *n* = 4) and non‐pCR (pre‐NAT_non‐pCR, *n* = 12; C3D1_non‐pCR, *n* = 12), respectively. (D) Venn diagram shows the intersection of proteins with significant changes at C3D1 stage in both groups (pCR, *n* = 4; non‐pCR, *n* = 12). (E) MCP1 protein curves with significant changes in pCR group alone at C3D1 (pCR, *n* = 4; non‐pCR, *n* = 12). (F) IL‐4 protein curves with significant changes in non‐pCR group alone at C3D1 (pCR, *n* = 4; non‐pCR, *n* = 12). (G) KEGG enrichment analysis of 13 proteins. Five proteins (IL‐22 RA1, 4E‐BP1, CD6, MCP‐1, and MCP‐3) were significantly changed alone in the pCR group; among the 26 proteins with significant changes in non‐pCR alone, seven proteins (Beta‐NGF, IL‐10RB, IL‐17C, IL4, NT‐3, OPG, and UPA) showed an opposite trend in pCR group; among the seven proteins that underwent significant changes in non‐pCR and pCR patients at C3D1 stage, only one protein (SLAMF1) showed an opposite trend between the two groups. (H) Numbers of DEGs at C3D1 versus baseline in pCR (pre‐NAT_pCR, *n* = 4; C3D1_pCR, *n* = 4) and non‐pCR tumors (pre‐NAT_non‐pCR, *n* = 10; C3D1_non‐pCR, *n* = 10) and (I and J) Volcano plots of DEGs in the two groups. (K) Venn diagram shows the intersection of DEGs in different group at C3D1 stage (pCR, *n* = 4; non‐pCR, *n* = 10). (L) KEGG enriched immune‐related signaling pathways for the selected DEGs, which are within the regions represented by red and blue numbers in the K plot. (M) Integrated pathway analysis comparing proteomic (pCR, *n* = 4; non‐pCR, *n* = 12) and transcriptomic (pCR, *n* = 4; non‐pCR, *n* = 10) alterations at C3D1. (N) Ligand–receptor mapping of 13 potential signatures (identified from D) to immune‐cell origins, highlighting 6 candidates (4E‐BP1, CD6, MCP‐1, IL‐10RB, uPA, and SLAMF1) associated with B‐cell subsets. (O) Cross‐modal heatmap showing correlations between immune‐related DEGs and six proteins and their associated B‐cell subtypes (pCR, *n* = 4; non‐pCR, *n* = 10). C3D1, cycle 3 Day 1; DEG, differentially expressed gene; KEGG, Kyoto Encyclopedia of Genes and Genomes; NAT, neoadjuvant therapy; NF‐κB, nuclear factor kappa B; pCR, pathologic complete response.

RNA‐seq at C3D1 identified 697 differentially expressed transcripts in pCR tumors (503 up, 194 down) and 484 in non‐pCR tumors (244 up, 240 down) relative to baseline (Figure 4H–J). Venn mapping of C3D1 versus pre‐NAT DEGs (Figure 4K) defined four analytic subsets: (i) 481 genes uniquely upregulated in pCR; (ii) 187 genes uniquely downregulated in pCR; (iii) five genes upregulated in pCR but downregulated in non‐pCR; and (iv) three genes showing the inverse pattern (up in non‐pCR and down in pCR). These four subsets (481, 187, five, and three genes) were carried forward for immune‐related pathway enrichment and compared with the proteomic profiles (Figure 4L,M).

xCell deconvolution highlighted B‐cell compartments as the main on‐treatment signal (C3D1 vs. pre‐NAT) (Figure S5). In the non‐pCR group, total B cells were significantly reduced at C3D1 (*p *= 0.043) with nonsignificant downward trends in naive and memory subsets. In the pCR group, all B‐cell subsets showed nonsignificant upward trends, likely reflecting limited sample size. Next, we mapped the previous potential signatures from Figure 4D to cell types using public single‐cell references. Ultimately, only six candidates (4E‐BP1, CD6, MCP‐1, IL‐10RB, uPA, and SLAMF1) aligned with B‐cell lineages (Figure 4N), with their cross‐modal correlations illustrated in Figure 4O.

Proteomic changes were less pronounced at a later timepoint (post‐NAT vs. C5D1; Figure S6), suggesting that C3D1 may represent a useful exploratory window for assessing early immune and signaling adaptation during which therapeutic modulation may influence efficacy.

### 2.6 Longitudinal Proteomic Programs and Candidate Biomarkers

Finally, we investigated sequential proteomic and transcriptomic changes across all treatment stages. At the protein level, time‐series analysis identified 13 candidates showing sustained increases across treatment stages (Figure 5A), consistent with STEM‐type modeling. Integration with repeated‐measures ANOVA confirmed a subset of 11 proteins with significant or trend‐level phase–response interactions (Figure 5B). KEGG enrichment of these 11 dynamic proteins pointed to immune‐ and inflammation‐related pathways (Figure 5C). Representative trajectories for CD40 and MCP‐1 are shown (Figure 5D,E). Parallel RNA‐seq demonstrated stage‐dependent transcriptional changes in the same directions (Figure S7).

**FIGURE 5:**
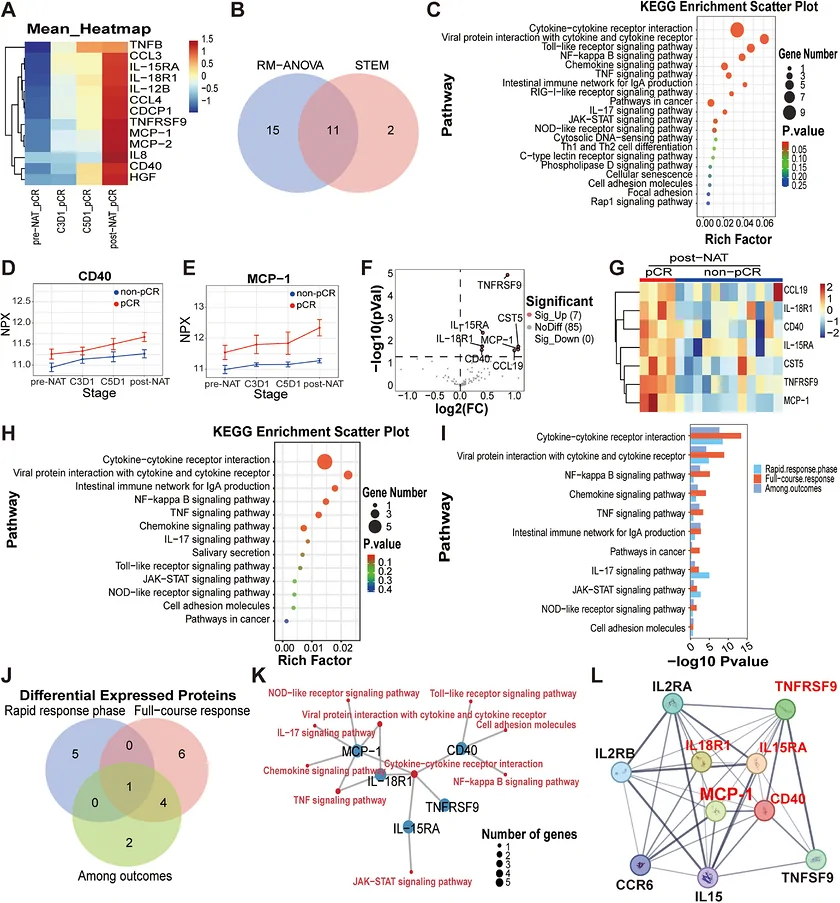
Continuous proteomic and transcriptomic dynamics across treatment phases. (A) Heatmap of time‐series clustering (STEM) showing 13 proteins with sustained increases across treatment (pre‐NAT, C3D1, C5D1, post‐NAT; pCR, *n* = 4; non‐pCR, *n* = 12). (B) Venn diagram integrating repeated‐measures ANOVA and STEM analysis, identifying 11 proteins with phase‐dependent changes, and (C) KEGG enrichment of these 11 proteins. Eleven proteins: CCL3, CCL4, CD40, HGF, IL‐12B, IL‐15RA, IL‐18R1, IL8, MCP‐1, TNFB, and TNFRSF9. (D and E) Longitudinal expression trajectories of CD40 and MCP‐1 across treatment stages (pCR, *n* = 4; non‐pCR, *n* = 12). (F and G) Volcano plot and heatmap of proteins between pCR (post‐NAT_pCR, *n* = 4) and non‐pCR (post‐NAT_non‐pCR, *n* = 12) patients at the post‐NAT stage and (H) KEGG pathways of these proteins. (I) Integrated KEGG pathway analysis of proteins identified from rapid‐response, full‐course, and posttreatment comparisons. Rapid‐response phase: pre‐NAT–C3D1; full‐course response: pre‐NAT–C3D1–C5D1–post‐NAT (pCR, *n* = 4; non‐pCR, *n* = 12); and among outcomes: post‐pCR (*n* = 4) versus post‐non‐pCR (*n* = 12). (J) Screening cross‐proteins by three‐way Venn diagram. One triple cross protein: MCP‐1; four double cross proteins: TNFRSF9, IL‐15RA, IL‐18R1, and CD40. (K) Pathway network map of five candidate proteins (two or more intersections of Venn analysis) and their enrichment pathways. (L) Protein–protein interaction network shows the proteins screened by Venn and the prediction of the protein with the strongest interaction with them. ANOVA, analysis of variance; C3D1, cycle 3 Day 1; C5D1, cycle 5 Day 1; KEGG, Kyoto Encyclopedia of Genes and Genomes; NAT, neoadjuvant therapy; pCR, pathologic complete response; STEM, Short Time‐series Expression Miner.

At the post‐NAT stage, volcano and heatmap analyses identified seven proteins—TNFRSF9, IL15RA, CD40, MCP‐1, CST5, NT‐3, and 4E‐BP1—with overall higher levels in pCR tumors compared with non‐pCR (Figure 5F,G). KEGG analysis based on the seven proteins is shown in Figure 5H. In addition, Figure 5I summarizes and integrates KEGG pathways involved in differentially expressed proteins from different groups (rapid response phase, full‐course response, and among outcomes), showing the key immune and inflammation‐related pathways involved, including cytokine–cytokine receptor interaction, viral protein–cytokine interaction, NF‐κB, chemokine, TNF and IL‐17 signaling pathways.

A three‐way Venn diagram (Figure 5J) identified MCP‐1 as the only protein shared across these three groups. TNFRSF9, IL‐15RA, IL‐18R1, and CD40 shared across full‐course and outcome analyses. We then mapped these five proteins to immune pathways to visualize regulatory linkages and inter‐protein relationships, revealing convergent functional connectivity via distinct pathways (Figure 5K). Further protein–protein interaction analysis placed the five key proteins within two immune modules—the TNF/TNFR costimulatory axis (TNFSF9–TNFRSF9, CD40) and the common γ‐chain cytokine axis (IL‐2/IL‐15 with IL2RA/IL2RB/IL15RA). Notably, MCP‐1 appeared to occupy a highly connected position, linking nodes across both modules and suggesting a bridging role between chemokine recruitment and costimulatory/cytokine signaling (Figure 5L).

In addition, in non‐pCR cases, mIF comparing pre‐NAT and post‐NAT showed a broad downward shift across multiple immune lineages, whereas CD8^+^ T‐cell density exhibited a nonsignificant upward trend (Figure S8A,B). M2/M1 ratio trended lower posttreatment; however, PD‐1^+^ CD8^+^T cells increased significantly, indicating greater exhaustion despite CD8 T cell abundance (Figure S8B).

## 3 Discussion

In this single‐arm, single‐center exploratory trial, we evaluated RC48 plus penpulimab as neoadjuvant therapy for patients with early or locally advanced HER2‐low breast cancer, most of whom had HR‐positive disease. Among 20 enrolled patients, the ITT pCR rate was 20.0%, and among 16 protocol‐evaluable patients, the pCR rate was 25.0%. Because no formal sample size calculation was performed and the confidence intervals were wide, these estimates should be considered preliminary and descriptive. The study was designed to generate clinical and translational hypotheses rather than to establish definitive efficacy. Given differences in patient populations, treatment regimens, and pCR definitions across studies, direct numerical comparisons with historical estimates should be avoided.

In prior reports, HER2‐low tumors—particularly HR‐positive subtypes—have generally shown limited responsiveness to standard chemotherapy [28]. Given differences in patient populations, treatment regimens, and pCR definitions across studies, direct numerical comparisons with such historical estimates should be avoided, and our pCR rate is presented descriptively rather than as a benchmark of relative efficacy.

Although T‐DXd has shown substantial activity in metastatic HER2‐low disease [6], early neoadjuvant experience with ADC monotherapy, such as TRIO‐US B‐12 TALENT, has reported lower pCR rates in the curative‐intent setting. Our study explores a conceptually different ADC–ICI combination strategy in a distinct patient population, and the observed pCR rate should not be interpreted as a head‐to‐head comparison with ADC monotherapy; rather, it provides preliminary, hypothesis‐generating support for further evaluation of ADC plus checkpoint inhibition in randomized trials. This concept is supported by large Phase III immunotherapy trials such as KEYNOTE‐756 [29] and CheckMate 7FL [30], which suggest potential benefit of immune checkpoint inhibitors in ER‐positive/HER2‐negative breast cancer, though efficacy in Asian subgroups has been less pronounced. However, given the exploratory design and limited sample size, our findings should not be viewed as definitive validation of a chemotherapy de‐escalation strategy; rather, they support further evaluation in randomized studies designed to establish comparative efficacy, safety, and tolerability.

The safety profile of the combination was consistent with the known toxicities of each agent, with no unexpected adverse events or treatment‐related deaths. Common events included pruritus, liver enzyme elevations, and constipation, while Grade ≥3 toxicities were infrequent. These results support the feasibility of further clinical evaluation of this regimen in the neoadjuvant setting.

Beyond clinical endpoints, this study generated exploratory translational hypotheses. At baseline, CCL19 and M2/M1 macrophage ratio appeared to be associated with pCR, consistent with a more immune‐active and less immunosuppressive TME. We therefore constructed an exploratory two‐feature BRPscore based on these variables. However, neither predictor reached statistical significance in the multivariable model, and the score was derived from only 12 to 13 biomarker‐evaluable patients with very few pCR events. Although the BRPscore showed good apparent discrimination (AUC = 0.8889), internal validation by LOOCV yielded a substantially lower AUC of 0.6667, and bootstrap optimism correction gave an optimism‐corrected AUC of 0.8375. These results indicate substantial optimism and instability, likely attributable to the limited sample size and the small number of pCR events. Consequently, the discriminative ability of the BRPscore remains uncertain, and for now it should be viewed as a hypothesis‐generating candidate biomarker signature rather than a validated predictive model. Larger independent cohorts are needed to determine whether CCL19 and the M2/M1 macrophage ratio can reproducibly predict response to ADC–ICI therapy in HER2‐low breast cancer.

CCL19 (also known as ELC/MIP‐3β) is a CC‐family chemokine that, together with CCL21, is the principal ligand of CCR7. It is produced largely by fibroblastic reticular cells and other stromal elements of secondary lymphoid organs, where it directs the homing of mature DCs, naive and central‐memory T cells, and certain B‐cell subsets into T‐cell zones, thereby orchestrating antigen presentation and the priming of adaptive immunity [31, 32]. Within the TME, CCL19–CCR7 signaling is one of the core axes driving the assembly and maturation of tertiary lymphoid structures (TLS), which serve as in situ hubs for tumor‐antigen‐specific immune activation [33]. In breast cancer, several lines of evidence have established CCL19 as a favorable immunomodulatory determinant rather than a tumor‐promoting factor: integrative analyses of TCGA cohorts have shown that high intratumoral CCL19 expression correlates with an immune‐active TME signature, increased infiltration of CD8^+^ T cells and proinflammatory macrophages, and improved overall survival [34]. More recently, a CCL19‐expressing DC population has been identified as a key correlate of TLS formation, lymphoid aggregation, and clinical benefit from anti‐PD‐L1 therapy in TNBC, with both circulating and intratumoral CCL19 levels tracking with response and survival under immunotherapy [35]. These data are consistent with our exploratory observation that higher baseline CCL19, together with a lower M2/M1 ratio, may indicate a TME more permissive to adaptive immune activation. In this small biomarker‐evaluable subset, pCR cases were observed within the BRPscore‐High group; however, this finding requires validation and should not be interpreted as evidence of clinical utility. These findings suggest that pretreatment immune contexture may inform future biomarker development in HER2‐low disease.

Dynamic molecular profiling suggested that biological divergence may occur during the rapid‐response phase (C3D1). During this window, MCP‐1 showed recurrent associations across baseline, early on‐treatment, and longitudinal analyses, suggesting that it may represent a candidate pharmacodynamic marker linking chemokine recruitment with costimulatory and cytokine signaling pathways. However, its role as a treatment–response biomarker or therapeutic target remains speculative and requires mechanistic validation.

MCP‐1 (CCL2), the founding member of the CC chemokine family, signals primarily through CCR2 and serves as the dominant chemoattractant for classical (CD14^+^CD16^−^) monocytes, while also recruiting memory T cells, NK cells, and DC precursors to sites of inflammation and tissue remodeling [36, 37]. Functionally, this recruitment axis may influence T‐cell activation indirectly by shaping the pool of antigen‐presenting myeloid cells and DC precursors within the TME, thereby linking chemokine changes with adaptive immune activation. Within tumors, MCP‐1 is produced by malignant cells, cancer‐associated fibroblasts, endothelial cells, and tumor‐infiltrating myeloid cells themselves, generating an autocrine or paracrine loop that shapes the myeloid landscape [37, 38]. In breast cancer specifically, MCP‐1 expression in the primary tumor and elevated circulating levels have been linked to tumor‐associated macrophage density, advanced clinical stage, early recurrence, and poor prognosis, and preclinical models have demonstrated a causal contribution of the CCL2–CCR2 axis to lung and brain metastasis through recruitment of protumor macrophages and myeloid‐derived suppressor cells [38, 39, 40]. Importantly, MCP‐1 is not merely a recruiter: it interacts with costimulatory programs and cytokine networks (e.g., GM‐CSF, TNF‐α, IL‐6 and CD80/CD86 axes) that govern macrophage polarization between immunosuppressive M2‐like and immunostimulatory M1‐like states, positioning it at the interface of innate cell trafficking and adaptive immune activation [41, 42]. This dual role may explain its context‐dependent behavior in oncology—protumorigenic when engaging immunosuppressive macrophages, but potentially favorable when therapy‐induced MCP‐1 surges remodel the myeloid compartment toward an antigen‐presenting, T‐cell‐supportive phenotype [37, 41].

Taken together, these exploratory findings suggest a potential framework for future studies: (i) prospective evaluation of baseline immune‐contexture scores such as BRPscore, and (ii) longitudinal assessment of candidate pharmacodynamic markers such as MCP‐1, TNFRSF9, IL‐15RA, and CD40. Such an approach could help generate biomarker hypotheses but requires validation before being applied to patient selection or treatment adaptation.

Several limitations warrant consideration. First, this was a single‐arm, single‐center, exploratory trial; the absence of randomization and a concurrent control arm precludes causal attribution of the observed pCR and ORR to the study regimen and prevents formal comparison with historical chemotherapy or chemotherapy plus immunotherapy regimens [43]. Second, cross‐trial comparisons with external datasets—including KEYNOTE‐522, IMpassion031, KEYNOTE‐756, CheckMate 7FL, and TRIO‐US B‐12 TALENT—are confounded by heterogeneity in patient selection, HR status composition, HER2 definitions, backbone chemotherapy, pathologic assessment, and pCR definitions; numerical differences in response rates therefore cannot be equated with true differences in treatment effect, and our results should not be construed as evidence of superiority or of the feasibility of chemotherapy de‐escalation relative to approved regimens [44]. Third, the modest sample size (*N* = 20 enrolled, *N* = 16 evaluable) limits statistical power and renders subgroup analyses exploratory only, whereas long‐term endpoints (invasive disease‐free survival [iDFS], EFS, OS) remain immature. Fourth, the BRPscore and the dynamic MCP‐1 signal were derived from a small biomarker subset within the same cohort; their reported performance is likely optimistic and requires prospective external validation together with mechanistic studies before any clinical application. Accordingly, all efficacy claims in this study are presented as hypothesis‐generating findings intended to inform the design of future randomized Phase II/III trials rather than as evidence of clinical benefit.

## 4 Methods

### 4.1 Study Design and Patient Population

This was a single‐arm, single‐center exploratory clinical trial (ClinicalTrials.gov identifier: NCT05726175) evaluating RC48 in combination with penpulimab as neoadjuvant therapy for patients with HER2‐low early or locally advanced breast cancer. The protocol was approved by the Ethics Committee of West China Hospital (approval number [2022 (1990)]) and conducted in accordance with Good Clinical Practice and the Declaration of Helsinki. All patients provided written informed consent before enrollment (August 2023 to August 2024).

Key eligibility criteria included women aged 18–75 years with histologically confirmed invasive breast cancer, ECOG performance status of 0–1, and clinical stage cT1N1–2 or cT2–4Nx with HER2 IHC 1+ or 2+/FISH−. Major exclusion criteria were inflammatory, metastatic, or bilateral breast cancer; prior antitumor therapy within 12 months; clinically significant cardiac disease; prior exposure to PD‐1/PD‐L1 or CTLA‐4 inhibitors; and a history of neurologic or psychiatric disorders, including seizure, dementia, or substance abuse.

### 4.2 Treatment and Study Schema

Patients received RC48 2.0 mg/kg intravenously plus penpulimab 200 mg intravenously every 3 weeks as neoadjuvant therapy for up to six cycles, followed by surgery. Serial biospecimens were collected at prespecified time points: tumor biopsy and blood before treatment (pre‐NAT), peripheral blood at C3D1 and C5D1, and tumor tissue and blood at surgery (post‐NAT). Paired tumor and blood samples were used for standard pathology and exploratory translational analyses, including Olink proteomics, IHC, mIF, and RNA‐seq.

### 4.3 Assessments

Radiologic tumor response was assessed by magnetic resonance imaging at protocol‐specified time points and evaluated according to Response Evaluation Criteria in Solid Tumors version 1.1.

Pathologic response was evaluated in surgical specimens after neoadjuvant treatment. pCR was defined as the absence of residual invasive cancer in the breast and regional lymph nodes, with residual ductal carcinoma in situ permitted (ypT0/is ypN0). Breast pCR was defined as ypT0/is irrespective of nodal status. RCB was calculated using the validated MD Anderson RCB method.

Safety was assessed in all patients who received at least one dose of study treatment. Adverse events were monitored throughout treatment and graded according to the National Cancer Institute Common Terminology Criteria for Adverse Events version 5.0, with the highest grade recorded for each event.

Exploratory biomarker analyses were performed using serially collected tumor and peripheral blood specimens, including assessments of PD‐L1 expression, TILs, immune‐cell populations, proteomic profiles, and transcriptomic features.

### 4.4 Study Endpoints

The primary endpoint was pCR in the ITT population, indicating no residual invasive cancer in the breast or regional lymph nodes after neoadjuvant therapy, with ductal carcinoma in situ permitted.

Secondary endpoints included bpCR, RCB score, ORR, disease control rate (DCR), iDFS, EFS, and safety. ORR was defined as the proportion of patients who achieved complete response (CR) or partial response (PR) as their best overall radiologic response according to the RECIST v1.1. DCR was defined as the proportion of patients who achieved CR, PR, or stable disease. iDFS was defined as the time from surgery to the first occurrence of invasive disease recurrence, second primary invasive cancer, or death from any cause. EFS was defined as the time from treatment initiation to disease progression precluding surgery, locoregional or distant recurrence, or death from any cause.

Exploratory endpoints included biomarker correlates of treatment response in blood and tumor tissue, including PD‐L1 expression, TILs, circulating tumor cells CTCs, and immune cell subsets.

### 4.5 Multiplex Immunofluorescence

mIF was performed using a predefined antibody–fluorophore panel by Shanghai KR Pharmtech (Shanghai, China). FFPE tissue sections (4 µm) were deparaffinized, rehydrated, and subjected to antigen retrieval with EDTA buffer (pH 9.0) or citrate buffer (pH 6.0). Immunostaining was performed on a Krast 600 platform (Kuoran Biotech, Shanghai, China), following automated standard protocols. Each round of staining involved incubation with primary antibodies (e.g. CD8, CD68, CD163, FOXP3, CD56, PANCK) and tyramide signal amplification (TSA)‐based fluorophores (Opal 480, 520, 570, 620, 690, TSA‐DIG 780). After each staining cycle, signal removal and reactivation were automatically performed to prevent cross‐reactivity. Nuclei were counterstained with DAPI (CST; catalog # 4083S, 1:1000). The primary antibodies and their respective concentrations and sources are detailed in Table S1.

Additional details regarding slide scanning, ROI selection, image segmentation, quality control, and quantitative analysis are provided in the Supporting Information: Methods.

### 4.6 Olink Proteomics and Cell‐Source Annotation

Circulating proteins were quantified using the Olink Target 96 Inflammation panel (Olink Proteomics AB, Uppsala, Sweden), which measures 92 inflammation‐related proteins using proximity extension assay technology. The complete list of analytes is provided in Table S2. Samples were processed according to the manufacturer's protocol, with internal extension and inter‐plate controls used for quality control and normalization. Protein abundance was reported as normalized protein expression values on a log2 scale.

To explore the putative cellular sources of candidate proteins, we analyzed the publicly available single‐cell RNA‐sequencing dataset GSE176078, which includes 26 primary breast cancer samples. Gene‐expression patterns of the corresponding protein‐encoding genes were evaluated across annotated cell populations using Seurat. Cell‐type associations were interpreted as exploratory transcriptome‐based inferences rather than direct evidence of protein secretion.

Additional details regarding the assay procedure, quality control, normalization, and single‐cell mapping are provided in the Supporting Information: Methods.

### 4.7 RNA Sequencing and Bioinformatic Analysis

RNA sequencing was performed using the Illumina NovaSeq X Plus platform. After quality control, sequencing reads were aligned to the human reference genome (Ensembl release 112) using HISAT2 (version 2.2.1), and transcript abundance was quantified using StringTie (version 2.1.6).

Differential gene‐expression analysis was performed using DESeq2 based on gene‐level count data, with edgeR used as a sensitivity analysis. Genes with a *p* value <0.05 and an absolute fold change ≥1.5 were considered differentially expressed. KEGG pathway enrichment analysis was performed using a hypergeometric test.

Relative enrichment of immune and stromal cell populations was estimated from the transcriptomic expression matrix using the xCell algorithm implemented through TIMER2.0. Enrichment scores were used for between‐group comparisons and were not interpreted as absolute cell proportions. Data visualization was performed using R (version 4.1.3) and ggplot2 (version 3.3.3).

Additional details regarding sequence processing, differential expression analysis, pathway enrichment, and immune‐cell deconvolution are provided in the Supporting Information: Methods.

### 4.8 Statistical Analysis

Given the exploratory, single‐arm design, no hypothesis‐based sample size calculation was undertaken. The protocol specified a target enrollment of 20 patients, for which no statistical rationale was prespecified.

The ITT and safety populations included all patients who received at least one dose of study treatment (*N* = 20). The PP population included patients who completed all six treatment cycles and underwent surgery (*N* = 16).

Analyses were primarily descriptive because of the exploratory, single‐arm study design. Continuous variables were summarized using appropriate measures of central tendency and dispersion, and categorical variables were summarized as counts and percentages. Two‐sided exact binomial 95% confidence intervals were calculated for key binary efficacy outcomes.

Between‐group comparisons of proteomic and immune‐cell features were performed using the two‐sided Student's *t*‐test or Wilcoxon rank‐sum test, as appropriate. Paired longitudinal measurements were analyzed using the Wilcoxon signed‐rank test or repeated‐measures analysis of variance, as appropriate. Measurements from multiple regions of interest were averaged to obtain one patient‐level value before statistical testing.

Exploratory multivariable logistic regression was used to construct the BRPscore, and model discrimination was assessed using the area under the receiver operating characteristic curve. The cutoff for descriptive BRPscore stratification was selected by maximizing the Youden index in the development dataset. Internal model performance was explored using LOOCV and bootstrap optimism correction with 10,000 resamples. Given the small number of patients and pCR events, all biomarker and prediction‐model analyses were considered hypothesis‐generating.

Given the exploratory nature of the study and the targeted design of the Olink Target 96 panel, no adjustment for multiple comparisons was applied to the proteomic analyses. The reported *p* values were therefore considered nominal, and all proteomic findings were interpreted as hypothesis‐generating.

All tests were two‐sided, with *p* < 0.05 considered statistically significant. Analyses were performed using SPSS version 22.0 and R version 4.1.3. Additional statistical details are provided in the Supporting Information: Methods.

## 5 Conclusion

In summary, this exploratory study provides preliminary clinical and translational evidence supporting further investigation of neoadjuvant disitamab vedotin plus penpulimab in HER2‐low early breast cancer. Exploratory analyses identified CCL19, the M2/M1 macrophage ratio, and MCP‐1 as candidate biomarkers of response; however, the BRPscore was derived from a very small subset and showed potential overfitting (optimism). These biomarker findings should therefore be considered hypothesis‐generating only. They require validation in larger, independent randomized controlled trials before any clinical application.

## Author Contributions

Yuting Song, Lei Liu, Aaron Qi Zhang, and Xin Xie led the clinical trial implementation, including data acquisition, statistical analysis, figure generation, and manuscript drafting. Collection and assembly of data: Chunying Zhuang, Xiaorong Zhong, Yan Cheng, and Dan Zheng. Bing Wei served as the guide for pathological reading. Ping He, Xi Yan, Tinglun Tian, and Jie Chen contributed to patient recruitment and therapeutic management. Juanjuan Li assisted in data analysis of the RNA‐seq and Olink. Xiaoxiao Liu, Shibao Li, Hao Wang, and Ting Luo were responsible for the experimental design, data validation, and manuscript revision. All authors have read and approved the final manuscript.

## Funding

This study was supported by “Noncommunicable Chronic Diseases‐National Science and Technology Major Project” (No. 2023ZD0502300 [2023ZD0502303]), Key Program of the Science and Technology Bureau of Sichuan (No. 2024YFFK0352), as well as supported by “Sichuan Science and Technology Program” (No. 2025ZDZX0012); support was also provided by the National Natural Science Foundation of China (No. 32301278), Advanced Program of The Affiliated Hospital of Xuzhou Medical University (PYJH2024210), Xuzhou City Clinical Technical Backbone Training Program Project (2025211001), and Medical Research Project of Jiangsu Provincial Health Commission (H2023050).

## Ethics Statement

This study was approved by the Ethics Committee of West China Hospital, Sichuan University (approval number [2022 (1990)]), and was registered at ClinicalTrials.gov (identifier: NCT05726175). Written informed consent was obtained from all participants.

## Conflicts of Interest

The authors declare no conflicts of interest.

## Supporting information




**Supporting File 1**: mco270988‐sup‐0001‐SuppMat.docx

## Data Availability

The raw sequencing data have been deposited in the Genome Sequence Archive for Human (GSA‐Human), National Genomics Data Center, under accession number HRA013151 (BioProject: PRJCA045783), and are accessible at https://ngdc.cncb.ac.cn/gsa‐human/browse/HRA013151. As human genomic data, they are stored under controlled access in accordance with the Regulations on the Administration of Human Genetic Resources of China. Access can be requested via the “Request Data” function on the dataset page and is reviewed by the corresponding Data Access Committee (DAC No. HDAC006743).
