# Supplementary material for: Integrative Multiomics Profiling Reveals Immune Determinants of Response to Disitamab Vedotin Plus Penpulimab in HER2‐Low Breast Cancer
Source: MedComm (2020). 2026 Oct 4;7(10):e70988. doi: 10.1002/mco2.70988 (PMC13634177; doi:10.1002/mco2.70988)
Supplement: Supplementary file 1 [file MCO2-7-e70988-s001.docx]

**Integrative multi-omics profiling reveals immune determinants of response to disitamab vedotin plus penpulimab in HER2-low breast cancer**

Xiaoxiao Liu^1,2,#,*^, Yuting Song^1,#^, Lei Liu^3,4,#^, Aaron Qi Zhang^5,#^, Xin Xie^2,#^, Bing Wei^6^, Ping He^1^, Chunying Zhuang^1^, Juanjuan Li^2^, Xi Yan^1^, Tinglun Tian^1^, Xiaorong Zhong^1^, Jie Chen^7^, Yan Cheng^1^, Dan Zheng^1^, Shibao Li^4,*^, Hao Wang^8,*^, Ting Luo^1,*^

1 Institute of Breast Health Medicine, Sichuan Provincial Engineering Research Center of Intelligent Diagnosis and Treatment of Breast Diseases, Breast Center, Department of Medical Oncology, Cancer Center, West China Hospital, Sichuan University, Chengdu, Sichuan, 610041, China.

2 Department of Radiation Oncology, Cancer Center, Affiliated Hospital of Xuzhou Medical University; Cancer Institute, Xuzhou Medical University, Xuzhou, Jiangsu, 221000, China.

3 Department of physiology, School of Basic Medicine and Life Sciences, Xuzhou Medical University, Xuzhou, Jiangsu, 221000, China.

4 Department of Laboratory Medicine, Affiliated Hospital of Xuzhou Medical University, Xuzhou, Jiangsu, 221000, China.

5 Department of Anatomical and Cellular Pathology, State Key Laboratory of Translational Oncology, The Chinese University of Hong Kong, Hong Kong SAR, China.

6 Department of Pathology, West China Hospital, Sichuan University, Chengdu, Sichuan, 610041, China.

7 Division of Breast Surgery, Department of General Surgery, West China Hospital, Sichuan University, Chengdu, Sichuan, 610041, China.

8 Hepatobiliary Center of The First Affiliated Hospital, Nanjing Medical University; Research Unit of Liver Transplantation and Transplant Immunology, Chinese Academy of Medical Sciences, Nanjing, Jiangsu, China.

*Correspondence: Xiaoxiao Liu (drliuxx@126.com), Shibao Li (sdjnshlb@xzhmu.edu.cn), Hao Wang (wanghao_@njmu.edu.cn) and Ting Luo (luoting@wchscu.cn).

#These authors contributed equally: Xiaoxiao Liu, Yuting Song, Lei Liu, Aaron Qi Zhang and Xin Xie.

**Figure S1. Efficacy for the intent-to-treat (ITT) population and exploratory subgroup pCR rates analysis.**

(A) Longitudinal change in target-lesion size by treatment cycle (n = 20); each line represents one patient, colored by best overall response (PR, n = 9; SD, n =9; PD, n = 2). Dashed lines indicate standard RECIST thresholds.

(B) Waterfall plot of best percentage change from baseline (n = 20), color-coded by best overall response (PR, n = 9; SD, n =9; PD, n = 2); PD-L1 status (CPS strata) is annotated beneath each bar. Dashed lines indicate standard RECIST thresholds.

(C) Exploratory subgroup pCR rate analysis. Exact sample sizes (n) for each subgroup are as follows: Stage II (n = 9), Stage III (n = 7), LN+ (n = 14), LN- (n = 2), PDL1 CPS <1 (n = 7), PDL1 CPS >= 10 (n = 4), ER+ <10% (n = 2), ER+ >= 10% (n = 14), ER+ >= 10% & PDL1 CPS >= 10 (n = 3), HER2 2+ FISH- (n = 8), HER2 1+ (n = 8). Data labels represent the percentage rate.

**Figure S2. Differential proteins between pCR and non-pCR patients at baseline (pre-NAT).**

Violin plots show baseline expression of selected proteins in patients who achieved pCR (n = 4, red) versus those with non-pCR (n = 12, blue). Individual sample data points are overlaid on the plots. P-values are indicated above each comparison.

**Figure S3. Heat map of RNA comparison between pCR and non-pCR patients at baseline.**

Unsupervised hierarchical clustering of differentially expressed genes (DEGs) at baseline, comparing patients who achieved pCR (n = 4, red) versus those with non-pCR (n = 10, blue).

**Figure S4. Immune cell profiles at baseline (pre-NAT) in pCR and non-pCR patients.**

Box plots show selected immune cell populations (by mIF and IHC) in patients who achieved pCR (n = 3, red) versus non-pCR (n = 9, blue). Individual values of biological replicates are displayed as points overlaid on each box plot. P-values are indicated above each comparison.

**Figure S5. xCell deconvolution results and selected B-cell subsets (C3D1 *v.s.* pre-NAT).**

(A) Deconvolution results in pCR patients (pre-NAT, n = 4; C3D1, n = 4).

(B) Selected B-cell subsets in pCR patients at C3D1 (n = 4) versus pre-NAT (n = 4). (C) Deconvolution results in non-pCR patients (pre-NAT, n = 10; C3D1, n = 10).

(D) Selected B-cell subsets in non-pCR patients at C3D1 (n = 10) versus pre-NAT (n = 10). Individual values are overlaid on violin plots. P-values are indicated above each comparison.

**Figure S6. Proteomic dynamics during the late response phase (C5D1).**

(A) Differential protein statistics of pCR and non-pCR patients in the rapid response stage (pre-NAT to C3D1) and late response stage (C5D1 to post-NAT), respectively (n = 4 per timepoint for pCR; n = 12 per timepoint for non-pCR). Numbers above bars represent DEG counts.

(B) Differential protein heatmap of pCR (n = 4 per timepoint) and non-pCR (n = 12 per timepoint) patients in the late response stage (C5D1 to post-NAT).

(C) KEGG enrichment of differentially expressed proteins in C5D1 to post-NAT of pCR patients (n = 4).

**Figure S7. Longitudinal RNA changes across three treatment periods in pCR and non-pCR patients.**
(A) Numbers of significantly altered DEGs identified by repeated-measures ANOVA across three timepoints (pre-NAT, C3D1, C5D1) in pCR (n = 4 per timepoint) and non-pCR (n = 12 per timepoint) patients.

(B) Temporal trends of RNA changes based on STEM analysis in pCR (n = 4) and non-pCR (n = 12) groups.

(C) Venn diagram shows the intersection of DEGs among different groups.

(D) Heatmap of selected DEGs across timepoints (pre-NAT, C3D1, C5D1) within the regions represented by red (n = 4 per timepoint) and blue (n = 12 per timepoint) numbers in panel C.

(E) KEGG pathways enriched among selected DEGs in pCR and non-pCR patients.

**Figure S8. Immune-related cellular changes in non-pCR patients at baseline and after neoadjuvant therapy.**

(A) Heatmap of immune-related cells in non-pCR patients at pre-NAT (n = 9) and post-NAT (n = 9).

(B) Boxplots showing representative immune cell subsets in pre-NAT (n = 9) versus post-NAT (n = 9) non-pCR patients, highlighting an increase in exhausted CD8+ T cells (Tex(PD-1)) after treatment. Individual sample data points are explicitly shown over the boxplots. P-values are indicated above each plot.

**Supplementary Table 1.** Antibody optimization by multiplex immunofluorescence using the Opal fluorophores (Akoya Biosciences).

|  | **Antibody (Ab.)** | **Manufacturer** | **Catalog NO.** | **Antigen retrieval** | **Ab. Dilution** | **Fluorophore ( F.)** | **F. Dilution** |
| --- | --- | --- | --- | --- | --- | --- | --- |
| **panel1** | **CD20** | Maixin | KIT-0001 | PH6 | 1:1 | 480 | 1:100 |
|  | **CD4** | Maixin | RMA-0620 | PH9 | 1:1 | 570 | 1:100 |
|  | **CD11c** | Abcam | AB52632 | PH9 | 1:1000 | 690 | 1:100 |
|  | **CD21** | Maixin | RMA-0811 | PH9 | 1:1 | 520 | 1:100 |
|  | **CD23** | Bioss | BSM6045R | PH9 | 1:2000 | 620 | 1:100 |
|  | **CD86** | CST | 91882s | PH9 | 1:200 | TSA-DIG 780 | 1:100 |
| **panel2** | **CD8** | Maixin | RMA-0514 | PH9 | 1:100 | 480 | 1:100 |
|  | **CD68** | Maixin | KIT-0026 | PH6 | 1:1 | 570 | 1:100 |
|  | **CD163** | Maixin | MAB-0869 | PH9 | 1:1 | 690 | 1:100 |
|  | **FOXP3** | Maixin | MAB-10004 | PH9 | 1:1 | 520 | 1:100 |
|  | **CD56** | Maixin | MAB-0743 | PH9 | 1:1 | 620 | 1:100 |

**Supplementary Table 2. All 92 marker proteins included in Olink Target 96 Inflammation**

| **UniProt** | **Protein target** | **Abbreviation** |
| --- | --- | --- |
| P00813 | Adenosine deaminase | ADA |
| Q9Y653 | Adhesion G-protein coupled receptor G1 | ADGRG1 |
| Q15389 | Angiopoietin-1 | ANGPT1 |
| O15123 | Angiopoietin-2 | ANGPT2 |
| P05089 | Arginase-1 | ARG1 |
| Q16790 | Carbonic anhydrase 9 | CAIX |
| Q14790 | Caspase-8 | CASP-8 |
| Q92583 | C-C motif chemokine 17 | CCL17 |
| Q99731 | C-C motif chemokine 19 | CCL19 |
| P78556 | C-C motif chemokine 20 | CCL20 |
| P55773 | C-C motif chemokine 23 | CCL23 |
| P10147 | C-C motif chemokine 3 | CCL3 |
| P13236 | C-C motif chemokine 4 | CCL4 |
| Q9BZW8 | Natural killer cell receptor 2B4 | CD244 |
| P26842 | CD27 antigen | CD27 |
| P10747 | T-cell-specific surface glycoprotein CD28 | CD28 |
| P01730 | T-cell surface glycoprotein CD4 | CD4 |
| P25942 | Tumor necrosis factor receptor superfamily member 5 | CD40 |
| P29965 | CD40 ligand | CD40-L |
| P06127 | T-cell surface glycoprotein CD5 | CD5 |
| P32970 | CD70 antigen | CD70 |
| Q01151 | CD83 antigen | CD83 |
| P01732 | T-cell surface glycoprotein CD8 alpha chain | CD8A |
| O95727 | Cytotoxic and regulatory T-cell molecule | CRTAM |
| P09603 | Macrophage colony-stimulating factor 1 | CSF-1 |
| P78423 | Fractalkine | CX3CL1 |
| P09341 | Growth-regulated alpha protein | CXCL1 |
| P02778 | C-X-C motif chemokine 10 | CXCL10 |
| O14625 | C-X-C motif chemokine 11 | CXCL11 |
| P48061 | Stromal cell-derived factor 1 | CXCL12 |
| O43927 | C-X-C motif chemokine 13 | CXCL13 |
| P42830 | C-X-C motif chemokine 5 | CXCL5 |
| Q07325 | C-X-C motif chemokine 9 | CXCL9 |
| P07585 | Decorin | DCN |
| P01133 | Pro-epidermal growth factor | EGF |
| P48023 | Tumor necrosis factor ligand superfamily member 6 | FASLG |
| P09038 | Fibroblast growth factor 2 | FGF2 |
| P09382 | Galectin-1 | Gal-1 |
| O00182 | Galectin-9 | Gal-9 |
| P12544 | Granzyme A | GZMA |
| P10144 | Granzyme B | GZMB |
| P20718 | Granzyme H | GZMH |
| P14210 | Hepatocyte growth factor | HGF |
| P09601 | Heme oxygenase 1 | HO-1 |
| O75144 | ICOS ligand | ICOSLG |
| P01579 | Interferon gamma | IFN-gamma |
| P01583 | Interleukin-1 alpha | IL-1 alpha |
| P22301 | Interleukin-10 | IL10 |
| P29459_P29460 | Interleukin-12 | IL12 |
| P42701 | Interleukin-12 receptor subunit beta-1 | IL12RB1 |
| P35225 | Interleukin-13 | IL13 |
| P40933 | Interleukin-15 | IL15 |
| Q14116 | Interleukin-18 | IL18 |
| P60568 | Interleukin-2 | IL2 |
| O95760 | Interleukin-33 | IL33 |
| P05112 | Interleukin-4 | IL4 |
| P05113 | Interleukin-5 | IL5 |
| P05231 | Interleukin-6 | IL6 |
| P13232 | Interleukin-7 | IL7 |
| P10145 | Interleukin-8 | IL8 |
| P43629 | Killer cell immunoglobulin-like receptor 3DL1 | KIR3DL1 |
| Q13241 | Natural killer cells antigen CD94 | KLRD1 |
| P18627 | Lymphocyte activation gene 3 protein | LAG3 |
| Q9UQV4 | Lysosome-associated membrane glycoprotein 3 | LAMP3 |
| P01137 | Transforming growth factor beta-1 | LAP TGF-beta-1 |
| P13500 | C-C motif chemokine 2 | MCP-1 |
| P80075 | C-C motif chemokine 8 | MCP-2 |
| P80098 | C-C motif chemokine 7 | MCP-3 |
| Q99616 | C-C motif chemokine 13 | MCP-4 |
| Q29983_Q29980 | MHC class I polypeptide-related sequence A | MIC-A/B |
| P39900 | Macrophage metalloelastase | MMP12 |
| P09237 | Matrilysin | MMP7 |
| Q8WXI7 | Mucin-16 | MUC-16 |
| O76036 | Natural cytotoxicity triggering receptor 1 | NCR1 |
| P29474 | Nitric oxide synthase, endothelial | NOS3 |
| Q15116 | Programmed cell death protein 1 | PDCD1 |
| P01127 | Platelet-derived growth factor subunit B | PDGF subunit B |
| Q9NZQ7 | Programmed cell death 1 ligand 1 | PD-L1 |
| Q9BQ51 | Programmed cell death 1 ligand 2 | PD-L2 |
| P49763 | Placenta growth factor | PGF |
| P21246 | Pleiotrophin | PTN |
| Q02763 | Angiopoietin-1 receptor | TIE2 |
| P01375 | Tumor necrosis factor | TNF |
| Q9NP84 | Tumor necrosis factor receptor superfamily member 12A | TNFRSF12A |
| O75509 | Tumor necrosis factor receptor superfamily member 21 | TNFRSF21 |
| P43489 | Tumor necrosis factor receptor superfamily member 4 | TNFRSF4 |
| Q07011 | Tumor necrosis factor receptor superfamily member 9 | TNFRSF9 |
| O43557 | Tumor necrosis factor ligand superfamily member 14 | TNFSF14 |
| P50591 | Tumor necrosis factor ligand superfamily member 10 | TRAIL |
| O43508 | Tumor necrosis factor ligand superfamily member 12 | TWEAK |
| P15692 | Vascular endothelial growth factor A | VEGFA |
| P35968 | Vascular endothelial growth factor receptor 2 | VEGFR-2 |

**Supplementary Methods**

1. **Multiplex Immunofluorescence Image Acquisition and Quantitative Analysis**

Multiplex immunofluorescence-stained slides were scanned using a KR-HT5 multispectral scanner (KR Pharmtech, Shanghai, China) to generate QPTIFF images. Image acquisition was performed under controlled laboratory conditions at 10–30°C and a relative humidity of ≤70%. Slides were scanned using an Opal Polaris five- to seven-color imaging protocol with exposure times optimized for each fluorescence channel within a range of 0–100 ms. All stained slides were stored in the dark at 4°C and scanned within 2 weeks after staining to minimize fluorescence signal deterioration.

Single-stained control slides and unstained tissue sections were used to establish the spectral library and perform spectral unmixing. Images were initially reviewed using HT5 View and subsequently analyzed using inForm software (Akoya Biosciences, Marlborough, MA, USA) for tissue segmentation, single-cell segmentation, phenotypic classification, and marker quantification. Positive and negative control tissues were included in each staining batch to assess staining specificity and signal quality.

For each patient, regions of interest (ROIs) were selected from representative areas containing viable invasive tumor tissue by a breast pathologist or a trained analyst who was blinded to clinical response. Areas containing extensive necrosis, hemorrhage, tissue folding, edge artifacts, poor staining quality, or insufficient tumor tissue were excluded. Where sufficient viable tumor tissue was available, at least 10 non-overlapping ROIs were analyzed per sample.

Tumor and stromal compartments were segmented according to pan-cytokeratin expression. Single-cell segmentation was performed primarily on the basis of DAPI-positive nuclei, with cellular boundaries refined using available membrane and cytoplasmic signals. Marker-positivity thresholds were established using positive and negative control tissues, verified by visual inspection, and applied consistently to all samples stained and analyzed within the same experimental batch.

Immune-cell phenotypes were defined according to marker co-expression patterns. CD4⁺ T cells, CD8⁺ T cells, CD68⁺ macrophages, and CD68⁺CD163⁺ M2-like macrophages were quantified. Because the antibody panel did not include additional canonical M1 macrophage markers, CD68⁺CD163⁻ cells were operationally classified as M1-like macrophages for descriptive purposes. Accordingly, the M2/M1-like macrophage ratio was calculated as the density of CD68⁺CD163⁺ cells divided by the density of CD68⁺CD163⁻ cells.

Quantitative mIF measurements were expressed as cell densities or percentages, as appropriate for each immune-cell population. Values obtained from multiple ROIs were averaged to generate a single patient-level measurement for subsequent statistical analysis.

2. **Olink Proteomic Profiling and Single-Cell-Based Cell-Type Mapping**

Protein profiling was performed using the Olink Target 96 Inflammation panel (Olink Proteomics AB, Uppsala, Sweden) according to the manufacturer’s instructions. The panel uses proximity extension assay technology to quantify 92 inflammation-related proteins from a small sample volume. The complete list of proteins included in the panel is provided in Table S2.

Briefly, pairs of oligonucleotide-conjugated antibodies bind to their respective target proteins. When both probes bind in close proximity, the attached oligonucleotides hybridize and are extended by DNA polymerase to generate a target-specific DNA sequence. The resulting amplification products were quantified using a microfluidic real-time polymerase chain reaction platform.

Assay quality control and normalization were performed using internal extension controls and inter-plate controls to account for technical variation within and between assay runs. Protein abundance was reported as normalized protein expression (NPX), an arbitrary unit on a log2 scale, with higher NPX values indicating higher relative protein abundance. Samples and proteins that did not satisfy the predefined manufacturer-recommended quality-control criteria were excluded from the corresponding analyses, where applicable.

To investigate the putative cellular sources of proteins showing differential or clinically relevant expression patterns, we analyzed the publicly available single-cell RNA-sequencing dataset GSE176078, comprising 26 primary breast cancer samples. The dataset was processed using Seurat, and the original cell-type annotations provided with the published dataset were retained where applicable. Expression of genes encoding the candidate proteins was examined across malignant, immune, and stromal cell populations. Relative expression patterns were used to infer the cell populations most likely to express the corresponding genes.

Because these analyses were based on transcript abundance in an external single-cell dataset, the inferred cell-type associations were considered exploratory and were not interpreted as direct evidence of protein production or secretion in the present clinical cohort.

3. **RNA Sequencing, Differential Expression, and Immune-Cell Enrichment Analysis**

Total RNA extracted from tumor specimens was subjected to library preparation and sequencing on the Illumina NovaSeq X Plus platform. Raw sequencing reads were quality-controlled before alignment to the human reference genome based on Ensembl release 112 using HISAT2 (version 2.2.1). The aligned reads were assembled and quantified using StringTie (version 2.1.6). Transcript assemblies from individual samples were merged using gffcompare (version 0.9.8) to generate a unified transcript annotation where applicable.

Transcript abundance was expressed as fragments per kilobase of transcript per million mapped reads (FPKM) for expression visualization and downstream descriptive analyses. Differential gene-expression analysis was conducted using DESeq2 with the Wald test based on gene-level raw count data. edgeR was used as a complementary sensitivity analysis for pairwise comparisons. Genes meeting the prespecified threshold of P <0.05 and an absolute fold change ≥1.5 were classified as differentially expressed genes.

Differentially expressed genes were subjected to Kyoto Encyclopedia of Genes and Genomes pathway enrichment analysis using a hypergeometric test. Pathways with P <0.05 were considered enriched. Enrichment analyses were performed using OmicStudio tools.

The relative enrichment of immune and stromal cell populations was estimated using the xCell algorithm through the TIMER2.0 platform. The normalized gene-expression matrix was used as input, and breast invasive carcinoma was selected as the corresponding tumor type where required by the platform. xCell generates gene-signature-based enrichment scores for immune and stromal cell populations. These scores represent relative enrichment rather than direct estimates of cell fractions or absolute cell abundance.

All visualizations were generated using R (version 4.1.3) and the ggplot2 package (version 3.3.3).

4. **Detailed Statistical Analysis**

The intention-to-treat and safety populations included all enrolled patients who received at least one dose of study treatment. The per-protocol population comprised patients who completed all six planned neoadjuvant treatment cycles and underwent surgery.

Continuous variables were summarized using the mean and standard deviation or the median and range, according to their distributions. Categorical variables were presented as counts and percentages. Exact two-sided 95% confidence intervals for pCR, ORR, and RCB 0–1 rates were calculated using the Clopper–Pearson method.

For unpaired comparisons, normally distributed continuous variables were analyzed using the two-sided Student’s t test, whereas non-normally distributed variables were analyzed using the Wilcoxon rank-sum test. Paired pre-treatment and post-treatment measurements were compared using the Wilcoxon signed-rank test. For multiplex immunofluorescence analyses, measurements from multiple regions of interest were averaged to generate a single patient-level value; individual regions of interest were not treated as independent biological replicates.

Longitudinal protein-expression trajectories were evaluated using repeated-measures analysis of variance and time-series clustering, where applicable. Pathway enrichment was assessed using hypergeometric testing, and protein–protein interaction networks were generated using STRING.

An exploratory multivariable logistic regression model incorporating baseline CCL19 expression and the M2/M1-like macrophage ratio was used to construct the BRPscore. Model discrimination was summarized using the area under the receiver operating characteristic curve. For descriptive stratification, the cutoff was selected by maximizing the Youden index on the apparent receiver operating characteristic curve in the development dataset.

Internal model performance was assessed using leave-one-out cross-validation and bootstrap optimism correction with 10,000 resamples. The 95% confidence interval for the cross-validated area under the curve was estimated using stratified percentile bootstrap resampling. For optimism correction, the model was fitted in each bootstrap sample and evaluated in both the bootstrap sample and the original dataset. The mean difference between bootstrap and test performance was subtracted from the apparent area under the curve to obtain the optimism-corrected estimate.

Because the model was developed and internally assessed in the same small cohort, the cutoff and performance estimates were considered exploratory and were not intended for clinical decision-making. All statistical tests were two-sided, and P<0.05 was considered statistically significant. Analyses were conducted using SPSS version 22.0 and R version 4.1.3.
